# Effectiveness of Workplace Interventions for Improving Working Conditions on the Health and Wellbeing of Fathers or Parents: A Systematic Review

**DOI:** 10.3390/ijerph19084779

**Published:** 2022-04-14

**Authors:** Maiko Suto, Olukunmi Omobolanle Balogun, Bibha Dhungel, Tsuguhiko Kato, Kenji Takehara

**Affiliations:** 1 Department of Health Policy, National Center for Child Health and Development, Tokyo 157-8535, Japan; suto-ma@ncchd.go.jp (M.S.); 18mp201@slcn.ac.jp (B.D.); takehara-k@ncchd.go.jp (K.T.); 2 Graduate School of Public Health, St. Luke’s International University, Tokyo 104-0044, Japan; 3 Department of Social Medicine, National Center for Child Health and Development, Tokyo 157-8535, Japan; kato-tg@ncchd.go.jp

**Keywords:** fathers, parents, workplace intervention, health and wellbeing, work-life balance, job performance

## Abstract

Evidence on the effectiveness of workplace interventions for improving working conditions on the health and wellbeing of fathers is scarce. We reviewed studies on the effectiveness of various workplace interventions designed to improve working conditions for the health and wellbeing of employed fathers and their families. Randomized controlled trials (RCTs) and quasi-randomized controlled trials of workplace interventions applied to employees with the aim of improving working conditions of employed parents, compared with no intervention, other active arms, placebo, wait list, or usual practice were included. Studies involving only women were excluded. An electronic search of the Cochrane Central Register of Controlled Trials (CENTRAL), MEDLINE, EMBASE, PsycINFO, ERIC and SSCI was done for eligible studies. Studies were screened against predetermined criteria and assessment of risk of bias done using the Cochrane Handbook for Systematic Reviews of Interventions for RCTs and the Risk of Bias Assessment tool for Non-randomized Studies for non-RCTs. Of the 8229 records identified, 19 reports were included in this review: 14 reports from five RCTs and five reports from two quasi-RCT studies. The studies were conducted in four different countries among working populations from various sectors. Studies addressing issues related to improving working conditions of fathers alone were lacking. All included studies assessed intervention effects on various health-related outcomes, the most common being sleep disturbances and mental health outcomes. Interventions administered yielded positive effects on various health outcomes across all seven studies. All included studies had methodological limitations, while study designs and methodologies lacked comparability. Consequently, a narrative synthesis of evidence is provided. Based on our findings, providing workplace interventions for improving working conditions may improve some aspects of the health and wellbeing of employed parents, including fathers.

## 1. Introduction

In recent decades, gender roles have undergone some important changes. Fathers’ involvement in childcare has increased, and, in contrast to the traditional roles, men in many modern societies are required to be simultaneously provider, guide, friend, playmate, caregiver and nurturer [1]. The striking changes in the role of fathers within the family, demands of these multiple roles, and the tensions they sometimes produce, challenge men’s identities, relationships with their partners, the meaning and place of work in their lives and their sense of self as competent adults [1]. Another challenge is the need for men to balance the competing demands of caring for the family and paid work. Such incompatibility between the simultaneous demands of paid work and family roles, known as work–family conflict [2] have been shown to not only affect the ability to work, but physical health as well [3]. Some authors refer to the broader concept of work–life conflict, considering the general working population, and not just employees with families.

Prior research shows that a shift in role balance may result in negative effects within the family. For example, work–life conflict has been regarded as a work-related stressor, and is known to have a potentially negative impact on personal effectiveness, marital relations, parent-child relationships and even child development [4]. It has also been linked to decreased job and life satisfaction, as well as stress-related outcomes, such as psychological disorders, exhaustion and alcohol abuse [5]. Further, a recent review shows an association between work–life imbalance and general mental and physical health, health behavior, health service utilization and sleep [3]. Although results of gender-specific health outcomes remain inconclusive [3,6], a multi-country study shows that the association is very similar for men and women [7]. Specifically, a recent systematic review shows increased alcohol consumption as a consequence of work–family conflict and that family-to-work conflict is strongly associated with depression among fathers [3].

Given the growing number of dual career households [8,9], and the awareness of adverse effects that work–family conflict has on men and women [3,4,5], institutional initiatives promoted to support the reconciliation of work and family have increased rapidly in recent years [9,10,11]. However, many such initiatives tend to target mothers with young children, due to an increase in women’s labor force participation and quest for economic and social equality [9,10].

Workplace interventions have emerged as a set of comprehensive health promotion and occupational health strategies implemented at the worksite to improve employee health and work-related outcomes [12]. Work environment factors, including work time scheduling, work stress and work demands, are strongly associated with employees’ health and wellbeing [13,14] and there is evidence of the positive impact of workplace interventions on work ability [15], and physical and mental health [16]. However, these studies did not focus on men/fathers, as is the case with many public health interventions [17,18]. Nonetheless, the workplace where most fathers spend a lot of time may be an ideal setting to deliver interventions targeted at fathers. Given changing gender roles, it is important to identify and synthesize evidence on interventions among men/fathers. Furthermore, systematic reviews regarding evidence-based decision making on programs and policies for improving the health and wellbeing of fathers and their families are lacking. The main objective of the current study was to synthesize evidence on the effectiveness of workplace interventions designed to increase scheduling for stress and work schedule control, management of work demands, and leave and days off among working parents, so as to improve working conditions and positively impact the health and wellbeing of fathers and their families.

## 2. Materials and Methods

This systematic review was performed according to the protocol registered in the International Prospective Register of Systematic Reviews (PROSPERO 2020 CRD42020185894), and adhered to the Preferred Reporting Items for Systematic Reviews and Meta-Analyses (PRISMA) guidelines (see Supplementary Material S1) [19,20].

### 2.1. Search Strategy

We performed an electronic search of the Cochrane Central Register of Controlled Trials (CENTRAL), MEDLINE, EMBASE, PsycINFO, ERIC and SSCI, with databases searched from inception to March, 2020. The searches were related to three main keywords: (1) fathers, (2) workplace, and (3) working conditions. Consistency in the theme was applied by considering terminological and technical differences between the databases. Various synonyms and terms related to the main keywords were used. The search strategies did not impose limits on publication date, country, or region, and included only full text, peer-reviewed articles published in the English language. Three experienced librarians developed and executed the search strategy (Supplementary Material S2).

### 2.2. Inclusion and Exclusion Criteria

Prospective controlled studies, including randomized controlled trials and quasi-randomized trials of workplace interventions applied to employers and employees, with the aim of improving the working conditions of employed parents (focusing on fathers), compared with no intervention, other active arms, placebo, wait list, or usual practice were included. Controlled before-and-after studies, and interrupted time series, were also considered for inclusion. Searches were restricted to include studies with interventions targeting adults of child rearing age working in full- or part-time capacity regardless of whether they were parents or not. Studies where interventions focused on work flexibility (time schedule, place), work demands (working hours, workload), and leave and days off (paternity leave, childcare leave) were included, irrespective of the form of implementation e.g., targeted at individuals, groups, or online based programs. Reported outcomes of interest were related to health, social wellbeing, and job performance. The primary outcome measures were physical health (e.g., fatigue, sickness), mental health (e.g., depression, anxiety, stress), and general health (e.g., sleepiness) in fathers, mothers, and children. Secondary outcomes were social wellbeing, including quality of life (QOL), work–life balance (including time spent with children), couples’ relationships and parent-child relationships, social support, job performance, absenteeism and presenteeism.

We excluded studies where interventions only targeted mothers or women. Single arm studies, observational studies, including cohort studies without prospective control groups, cross-sectional, case-control studies and reviews were also excluded. The review PICO (Population, Intervention, Comparison, and Outcome) criteria are shown in Appendix A Table A1.

### 2.3. Study Selection

Three review authors (MS, OOB and BD) independently assessed all the titles and abstracts. After the titles and abstracts were screened, we retrieved full text of studies if it was included by at least one reviewer from the title and abstract screening. MS and OOB then independently screened the full texts for eligibility, using the same criteria applied for the title and abstract screening. At this stage, reasons for excluding studies were noted, and disagreements were resolved through discussion or consultation with a third author when required. The reference lists of included studies were examined to search for additional studies.

### 2.4. Data Extraction

Three review authors (MS, OOB and BD) independently extracted data from all included studies using a predesigned and pilot-tested data extraction form (see Supplementary Material S3). Discrepancies were resolved through discussion or by consulting with another co-author (KT). For studies with multiple reports, data extraction was done for each report separately and we planned multiple synthesis if our review prespecified outcomes were presented in multiple reports. The data extraction form included the following information: information on publication study design (study ID, year); study period; description of study population (e.g., fathers only or fathers and others, age, age of children); country; number of participants; type of workplace/industry; type of intervention (e.g., screening, counselling, health education etc.); delivery mode (e.g., individual, group, online); intervention characteristics (e.g., components, frequency and duration, provider); comparator characteristics (e.g., no intervention, wait list) and targeted outcomes (definition, measurement, estimates of effect with relevant statistics and follow-up period). Missing data were reported in the study results (risk of bias) table. When not provided in the report, we contacted authors of the included studies for information regarding employees with a child and proportion of male employees with a child living at home.

### 2.5. Risk of Bias Assessment

MS, OOB and BD independently assessed the risk of bias for each included study. For RCTs, risk of bias was assessed using criteria outlined in the Cochrane Handbook for Systematic Reviews of Interventions [21]. The following domains were assessed according to the Cochrane risk of bias checklist: random sequence generation, allocation concealment (also in the domain of selection bias), blinding of participants and personnel (performance bias). Supporting text for the judgment of risk of bias was provided for each assessment. Assessment of risk of bias for non-RCTs was done using the Risk of Bias Assessment tool for Non-randomized Studies (RoBANS) [22]. The following bias domains were assessed: selection of participants, confounding variables, measurement of intervention (exposure), blinding of outcome assessment (detection bias), incomplete outcome data (attrition bias, short term, or long-term, outcomes) and selective outcome reporting (reporting bias). Risk of bias for each domain was classified into three categories: low risk, high risk, or unclear risk of bias. Any disagreements were resolved through discussion with KT.

### 2.6. Data Synthesis

This review included studies using different study designs (RCTs and quasi-randomized trials). For dichotomous data, we planned to present results as summary risk ratios or odds ratios with corresponding 95% confidence intervals. In case of continuous variables, we planned to use the mean difference if outcomes were measured in the same way between studies, or standardized mean difference to combine studies that measured the same outcome, but used different assessment tools. We also planned to analyze relevant data from each study design separately and conduct subgroup analyses based on characteristics of participants (e.g., age, socio-economic status), type of intervention (e.g., duration), assessment points, and study design.

## 3. Results

### 3.1. Search Results

**Figure 1:**
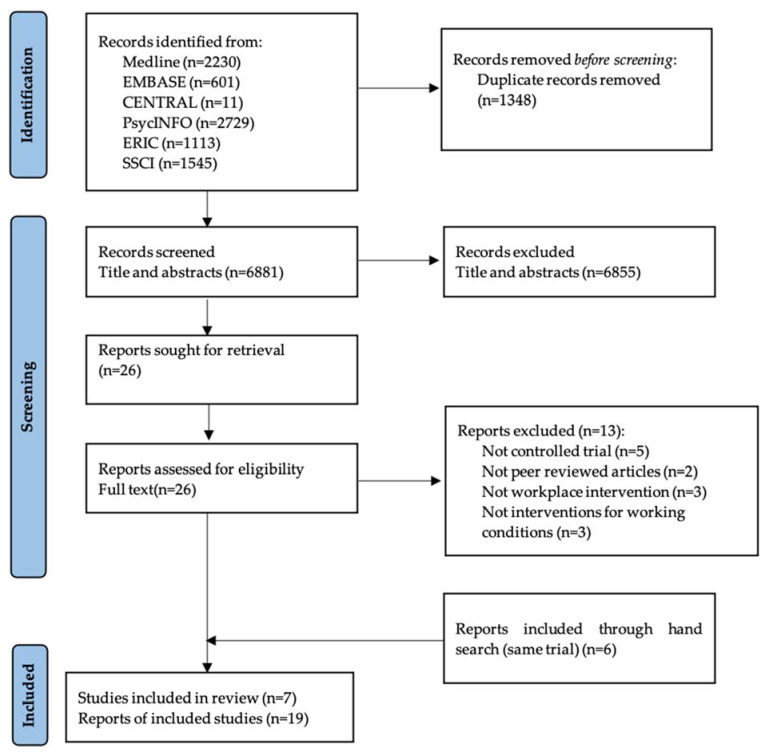
Flow diagram of the study selection process.

The search strategy applied to the six databases yielded 8229 records. After duplicates were removed, a total of 6881 citations remained for title and abstract screening. Full texts of 26 potentially eligible studies were retrieved for full text assessment and ineligible studies excluded with reason (see Supplementary Material S4). Thirteen records were excluded, while another six records identified from hand search of the references of included studies were added. Finally, seven studies reported in 19 publications fulfilled the inclusion criteria and were included in the review. Studies with multiple reports were presented as a single study in Table 1, showing characteristics of included studies; while outcome results from each study were presented as individual reports in Supplementary Material S5. Figure 1 shows the number of records identified, excluded and included.

### 3.2. Description and Characteristics of Included Studies

Five of the included studies (in 14 reports) [23,24,25,26,27,28,29,30,31,32,33,34,35,36] were RCTs (two cluster-RCTs), while the other two studies (in five reports) [37,38,39,40,41] used quasi-experimental controlled study design. Three studies [26,30,34] were carried out in Australia, two studies (11 reports) [23,25,27,28,29,31,32,33,39,40,41] in the United States and one study each in Sweden (three reports) [24,35,36] and Denmark (two reports) [37,38]. The studies all included working populations from various sectors, including healthcare, service and welfare, information technology, education, government and administration. Interventions targeted both male and female employees, and no study was identified which addressed issues related to improving the working conditions of fathers alone. The proportion of male employees in the included studies ranged from 10–60% (Supplementary Material S5). For example, in one study involving 840 employees, 66 were male and of these, only 32 (~4%) had a child living at home [37,38]. Alternatively, other studies included only working parents, wherein all male participants had a child aged 16 years or younger living at home [26,30,34]. The number of randomized participants across the included studies ranged from 45 to 3159 employees, with enrollment periods spanning between 2005 (or earlier) to 2014. Intervention duration lasted from eight hours to nine months, and follow-up periods of up to 18 months or less were reported. Most studies had a follow-up period of 12 months.

Intervention types varied across the studies and included reduced weekly working hours [24,35,36]; employee self-rostering (flexibility) [37,38]; workplace structural, social and cultural change process for managing work–family interface [23,25,27,28,29,31,32,33]; workplace parenting and work–family intervention [26,30,34] and employee assistance programs providing individualized counseling for identifying coping strategies for personal and professional stressors [39,40,41]. The included studies targeted health and social wellbeing-related outcomes, such as sleep quality, worry, stress, mental distress and somatic symptoms, problem behavior, work–life conflicts, work–family conflict, parental satisfaction, and dysfunctional parenting; and work-related outcomes, such as sick time taken, absenteeism, presenteeism and self-efficacy. Table 1 presents the characteristics of the included studies and the interventions they administered. Studies with multiple reports are listed under the same study ID in Table 1.

### 3.3. Risk of Bias Assessment

The overview of risk of bias of the included studies is presented in Table 2 and Appendix A Table A2 and Table A3. All studies had problems with various domains of study quality.

#### 3.3.1. Risk of Bias for RCTs

Overall, the RCTs were of low or unclear risk of bias. In terms of methods used for random sequence generation and allocation concealment, two of the RCTs [30,36] were assessed as being unclear because the details for both procedures were not reported. All the RCTs included in this review either did not provide information on, or lacked blinding of participants and personnel, and were assessed as having unclear or high risk of bias in this domain. Outcome assessment was based on participant self-reports using structured interviews, and was judged to be unclear for all included RCT studies. Martin and Sanders [30] had higher loss to follow-up in the control group than in the intervention group and it was assessed as having high attrition bias. Reporting bias was judged to be unclear in all included RCTs as neither trial registration nor study protocol were available, and due to inadequate description of outcomes in the text.

#### 3.3.2. Risk of Bias for Non-RCTs

In terms of selection of participants, Nunes, et al. [39] was assessed as having low risk of bias, whereas Albertsen, et al. [37] was judged to have a high risk of bias. Regardless, both studies were judged to have low risk of bias for confounding as the studies used statistical methods that controlled for major confounding factors in the analyses. Performance bias due to inadequate measurement of exposure was assessed as unclear in Albertsen, et al. [37] and Garde, et al. [38], but judged as high for Nunes, et al. [39], Richmond, et al. [40] and Richmond, et al. [41] due to data obtained through self-reports [39,40,41]. Detection bias caused by inadequate blinding of outcome assessment was assessed as unclear, given that outcome data was obtained through self-reports in the included non-RCT studies. Attrition bias was judged as unclear in Nunes, et al. [39], Richmond, et al. [40] and Richmond, et al. [41] due to large differences with respect to the number of eligible participants that enrolled in the study. For Albertsen, et al. [37] and Garde, et al. [38] with low attrition bias, missing outcome data was accounted for and balanced in numbers across intervention groups. Reporting bias was assessed to be unclear in included non-RCT studies, due to inadequate description of outcomes a priori.

### 3.4. Effects of Interventions

A total of five different intervention types were administered across the seven included studies. In this review, intervention effects were categorized into three groups: health (primary outcome), social wellbeing and job performance outcomes (secondary outcomes). The most common outcome category reported was health outcomes. Three individually randomized trials [26,30,34] assessed the effect of workplace parenting interventions on health, social wellbeing and job performance among a population that was 20–30% men. The two cluster RCTs assessed the effects of reduced working hours [24,35,36] and supervisor/employee training [23,25,27,28,29,31,32,33] on health and social wellbeing. Among non-RCTs, Albertsen, et al. [37] and Garde, et al. [38] assessed the effect of an intervention for employee self-rostering on health, social wellbeing, and job performance, while Nunes, et al. [39] assessed the effect of individualized counselling on health outcomes and job performance. The summary of intervention types and overall effects is presented in Table 2.

#### 3.4.1. Data Analysis

The studies varied with regards to participants, interventions, and outcomes. Additionally, each of the included studies employed a different questionnaire or method for outcome assessment, thus precluding the possibility of combining the summary estimates from individual studies in a meta-analysis. Consequently, we have provided a narrative synthesis of the findings from included studies, organized by outcome categories (related to health, social wellbeing, and job performance). Originally, we planned to focus on workplace intervention studies which involved fathers alone, but we broadened the scope of our study to include parents in general due to lack of studies on fathers only. Where possible, we report outcomes for fathers separately. However, intervention impact on parents in general, and child(ren), is also reported.

#### 3.4.2. Effectiveness of Workplace Interventions for Improving Working Conditions on Health-Related Outcomes

All the included studies [23,24,25,26,27,28,29,30,31,32,33,34,35,36,37,38,39,40,41] assessed intervention effects on various health-related outcomes. There were no negative effects reported in all seven studies. The most common health-related outcomes were sleep disorders, sleep duration and quality, and various mental health disorders. Additional health outcomes included stress biomarker cortisol awakening response levels, need for recovery, somatic symptoms, and workplace related stress. Interventions administered yielded significant positive effects across all seven studies. Details of findings from individual studies with statistical significance is presented in Supplementary Material S5. Schiller, et al. [35] (cluster RCT), which included 580 employees (24% men) working full-time from a total of 33 workplaces across four different public work sectors, evaluated the impact of a 25% reduction of weekly work hours with retained salary for 18 months on sleep, sleepiness, and perceived stress. Compared with the control group, the intervention group had improved sleep quality and sleep duration, and displayed reduced levels of sleepiness and perceived stress on workdays and days off. Specifically, men were shown to have benefitted more from worktime reduction, as compared with women, while male and female employees with children living at home reported somewhat lower levels of perceived stress on workdays, as compared to those without children. No statistically significant improvements were found in sleep duration on days off and daytime sleep among all employees [35,36] and time-use patterns in relation to the intervention were similar for men and women [36]. Four reports from an RCT involving 1171 US employees across 56 study groups working at an information technology firm [23,29,32,33], and randomized to intervention group or usual practice, examined the impact of a social and organizational change process on sleep duration and quality. The study findings showed that a subset of the employees who provided actigraphy recordings at baseline and at 12-month follow-up achieved increased sleep duration and reduced sleep insufficiency relative to the control group employees [29,33]. Similarly, findings by McHale, et al. [32] suggest the beneficial effect of the workplace intervention on sleep in employees’ adolescent children; whereby the intervention might serve to buffer youth from age-related declines in healthful sleep patterns. Further, Almeida, et al. [23] reported that in another subset of 94 employed parents, the intervention was effective in enhancing employees’ biological stress physiology, particularly during opportunities for recovery. Participants from two individually randomized controlled trials conducted in Australia to evaluate the efficacy of the Workplace Triple-P Positive Parenting Program were found to report lower levels of stress, depression and anxiety [26,34].

Similar findings were also reported among non-RCTs included in this review. In Denmark, introduction of self-rostering among shift workers using an IT-based tool resulted in improved health in terms of less need for recovery, better sleep, fewer somatic symptoms, and less mental distress [38]. In a separate quasi-experimental study conducted in the US among a diverse employee base, employees (male and female) on Employee Assistance Programs (EAP) were reported to have statistically significantly reduced symptoms of depression and anxiety compared with their matched controls, while no significant differences were reported with regards to alcohol use [40]. Further, EAP service users with problems producing moderate use of sick leave were found to have a steeper decline in sick leave usage compared to matched non-EAP service users [39]. The intervention was found to be less effective in reducing levels of sick leave for employees experiencing a high number of sick leave hours [39].

#### 3.4.3. Effectiveness of Workplace Interventions for Improving Working Conditions on Social Well-Being

Five RCTs (in 14 reports) [23,24,25,26,27,28,29,30,31,32,33,34,35,36] and one non-RCT study (in two reports) [37,38] assessed intervention effects on employee social wellbeing. The social wellbeing outcomes reported included work–life balance, marital conflict, parent-child relationship, parental satisfaction, and parenting efficacy. None of the studies reported quality of life outcome measures. All six studies found positive intervention effects, or no statistically significant difference, between the intervention and control groups. In a cluster RCT study among full time employees within the public sector in Sweden, reduced working hours had positive effects on negative emotions, work intrusion on private life [24], and work–life balance, whereby more time was spent on free-time activities within the intervention group, as compared with the control group [36]. In addition, time use patterns with regards to the intervention were similar for men and women as well as for those having children living at home and those who did not [36]. Other studies reported similar improvements in work–life balance through reducing work–family conflicts following implementation of different workplace interventions [26,27,28,37]. Specifically, statistically significant improvements in employees’ work–family conflict and family time adequacy, and larger changes in schedule control and supervisor support for family and personal life were found among fathers following an intervention to reduce work–family conflict [27]. Also, improvement in parent-child relationship was reported by Davis, et al. [25] and McHale, et al. [31] in a cluster RCT done in the US among an employee subgroup with children aged 9–17 years of age who participated in another workplace intervention to promote employee schedule control [25,31]. Of the 93 participants, 55% were fathers, and parents in the intervention group exhibited statistically significantly higher parent-child shared time, although the intervention effect was less in fathers [25]. Regardless, employee attendance of the majority of the intervention sessions resulted in more positive parent-child relationships, relative to employees in the control group [31]. Three individually randomized studies in Australia, involving up to 273 employees randomized to either a workplace parenting intervention or wait list control condition, all reported improvements in various domains of family functioning and parenting efficacy relative to the wait list control condition [26,30,34]. Decrease in marital conflict was reported in one study (non-RCT) involving 784 shift workers that implemented self-rostering [37]. Albertsen, et al. [37] was the only study to report a negative effect, wherein a subgroup of employees considered the flexibility intervention as employee unfriendly, and reported problems combining work and private life demands.

#### 3.4.4. Effectiveness of Workplace Interventions for Improving Working Conditions on Job Performance

Four studies (seven reports) [24,26,30,34,38,39,41] examined various workplace intervention effects on job performance outcomes. Three individual level intervention studies involving male and female employees done in Australia yielded statistically significant effects on job satisfaction, workplace commitment [34] and self-efficacy at work pre- and post-intervention [26,30]. In a cluster RCT study conducted in Sweden and involving full-time employees, reduced working hours had positive effects on time use patterns whereby workload decreased [36], and on job demand, manager support and intrusion on private life [24]. In Albertsen, et al. [37], a non-RCT study involving employees from 28 worksites in Denmark, an intervention designed to optimize personnel resources yielded a negative effect on work–family balance, due to much unpredictability and variability in working hours [37], while the intervention effect (in one of the three intervention type) caused more employees to consider changing job [38]. Nunes, et al. [39] and Richmond, et al. [41] involved employees from across 19 departments of the state public sector in the US to test an employee assistance program and measured sick leave usage and absenteeism using objective measurement. A statistically significant reduction in sick leave usage [39] and absenteeism [39,41] was reported among male and female employees in the intervention group compared to control group employees.

## 4. Discussion

This review on workplace interventions for improving working conditions sought to synthesize evidence on the effect of workplace interventions on the health and wellbeing of employed fathers and their families. Our findings show that providing interventions for improving working conditions in workplaces is promising for improving some aspects of the health and wellbeing of employed parents in general, and their children. Also, our review shows the need for research on the effectiveness of workplace interventions among men/fathers. We included only experimental (or quasi-experimental) studies with intervention components aimed at domains of work demands, work flexibility and/or leave and days off for working parents in this review. The most frequently evaluated intervention was a workplace parenting program that involved managing the work–family interface. The seven studies in this review included five RCTs (two cluster-RCTs) and two non-randomized controlled trials. Only one of the included studies was published earlier than the year 2010, while all the studies had problems with various domains of study quality.

All the included studies reported health-related outcomes. The most common health outcomes were related to sleep disturbances and psychological distress. Of the seven studies included in this review, three (in 13 reports) [23,24,25,27,28,29,31,32,33,35,36,37,38], that assessed sleep outcomes, reported statistically significant positive effects in various aspects of sleep timing, quality, and duration; while all the studies reported improvements in psychological distress following different workplace interventions. Among adults, work schedules (including shift work and long hours) are among the reasons for sleep loss [42] and the association between work time control and sleep disturbances has been established [43]. Similarly, we found evidence to support the association between interventions involving reduced working hours, or increased work flexibility, and improved sleep outcomes.

In relation to the secondary outcomes, six of the seven included studies reported on social wellbeing-related outcomes, including work–life balance, couple and/or parent-child relationship and social support; while only four examined outcomes related to job performance. Among social wellbeing outcomes, statistically significant effects were reported in relation to work–life balance [24,26,27,36,37], parent-child relationship [25,31] and social support [27]. Significant improvements were also reported in job performance outcomes including work efficacy [26,30], work commitment [34], absences from the worksite and improvements in productivity when at work [41]. Although the interventions varied across the studies, they were worker-oriented, increased flexibility and control for the worker and targeted managing the work–family interface.

Our review planned to focus on workplace interventions involving fathers. However, no studies were found that targeted fathers only. Male participation in the included studies ranged from 10% to 60%, while only three reports conducted subgroup analysis among men [27,35,36]. Consequently, the foreground of our findings did not include fathers alone. Several studies have also shown low male participation in workplace health promotion [44] and disease prevention programs [45]. A systematic review showed that participation in health promotion interventions at the workplace varies greatly between male and female employees, with higher participation among female workers [44]. Although there is some overlap in factors associated with participation in workplace health promotion programs between men and women [46], recruitment of men is shown to be improved with trial-specific training of personnel [45,47]. Another factor to consider for male participation is age. The ages of employed parents in the included studies ranged from 20–65 years old. Compared to older men, younger men have lower rates of health-seeking behavior [47], which may also have contributed to the male participation rates from included studies. A recent meta-analysis on the relationship between gender and work–family conflict concluded that men and women show similarities in their reports of work–family conflict [48]. However, there is still insufficient awareness concerning work–family conflict in fathers, especially in view of men’s changing roles. Workplace interventions have the potential to reach large groups, and the worksite is promoted as a setting for health promotion programs [12]. However, in order to improve health outcome in men and lessen the negative consequences associated with work–family conflict, recruiting strategies need to be tailored to the preferences of men to encourage participation

The certainty of the evidence presented in this narrative review may be weakened due to methodological variation and differences in the methods of outcome assessment. We had planned to explore further possible differences in intervention effect based on participant characteristics, such as age and socio-economic status, intervention duration, assessment points and study design. However, no subgroup analysis was done. Such variations in outcomes reported on a particular topic precludes synthesis of evidence, thus making it difficult to draw policy-oriented conclusions. Therefore, a core set of outcomes to be considered in workplace intervention research is recommended. Furthermore, none of the included studies analyzed intervention effect by income level or socio-economic status, while work sector was only very broadly mentioned to describe participant characteristics. Although some included studies stated the involvement of single and non-biological parents in their intervention programs, no study conducted any analysis within this sub-group. Single and non-biological parents face unique problems and may have different experiences than participants with partners or biological parents. Therefore, interventions aimed at improving the health and wellbeing of employed fathers within this sub-group is recommended in future studies.

Our review has several limitations. Heterogeneity was high among studies included in this review. Differences in intervention types, and variations in study design, setting and population limited comparability. Also, in some studies that included both parents and childless employees, it was not clear if all included male employees had children, neither could we ascertain that those employees with children were not mostly female. Therefore, derivation of general recommendations specific to fathers is not possible. Regardless, participants came from diverse occupational groups and the various analyses controlled for gender. Thus, valuable information could still be derived from the included studies. Also, inclusion of study designs other than RCTs is important in evaluating effects of complex interventions, such as workplace health promotion interventions. Given the focus on fathers, their health and wellbeing, keywords used in our search strategy to identify eligible studies may have been narrow and inadvertently excluded publications in occupational health that may have included fathers. However, searches and inclusion criteria were comprehensive enough and considered studies including both parents. Further, the search strategy was applied to major electronic databases that facilitated a more evidence-based approach to the literature search. Attempt was made to identify and include all relevant research articles, but we may have missed important contributions by including only full text articles published in the English language. Additionally, all included studies were from high-income countries, restricting the generalizability of our findings.

## 5. Conclusions

Based on the findings presented in this narrative synthesis, it is possible that interventions for improving working conditions in workplaces are likely to improve some aspects of the health and wellbeing of employed parents. However, improvements specifically for fathers could not be confirmed through this review, due to the lack of details regarding male employees in the included studies. Future research is needed to examine the effect of interventions on improving the working conditions of fathers in relation to fathers’ health and wellbeing and how this can lead to family benefits.

## Figures and Tables

**Table 1:** Characteristics of included studies.

Study ID	Study Period	Country	Design (Unit of Allocation)	Population Description Type of Workplace Age Range, Gender	Intervention Description Provider Follow-Up Period	Resources Economic Impact	Comparator	Outcomes Reported and Scale Used		
								Health	Social Wellbeing	Job Performance
Schiller 2017—Reduced weekly working hours										
Schiller, et al. [35], Schiller, et al. [36], Barck-Holst, et al. [24]	February 2005 November 2006	Sweden	Cluster-randomized controlled trial (workplace *n* = 33) Intervention-*n* = 17 involving 500 participants; control-*n* = 16 involving 419 participants)	Employees in four different working sectors: social services, technical services, care and welfare, and call center. Almost one third were shift workers, and about half of the participants had children living at home. Age range: 20–65 ~25% male (subgroup analysis done by gender)	25% reduction of weekly work hours (according to the employers’ time recording data over 14 months) Follow-up: 9 and 18 months	Participants retained their salaries and the workplaces obtained funding for recruiting more staff in order to avoid an increase in workload for the employees	No intervention	Employee Sleep; sleep length, sleepiness (Karolinska Scale) sleep quality (SSQ) worry and stress (at bedtime) perceived stress (Schiller 2017)	Employee Amount of time spent on recovery activity (Schiller 2018); demand, control and social support, manager support, coworker support, work intrusion on private life, private life intrusion on work (Barck-Holst 2015)	
Albertsen 2014—Self-rostering (Flexibility)										
Albertsen, et al. [37], Garde, et al. [38]	October 2008–October 2009	Denmark	Quasi-experimental intervention study (9 organizations with workplaces *n* = 28) Intervention *n* = 14; control *n* = 14 840 participants returned the questionnaire at baseline	Shift workers Mainly hospitals and psychiatric homes. Over half of participants had children living at home. Mean age 42 years ~10% male participants	Implementation of IT-based tools for self-rostering. (New work schedule based on the preferences of the employees and the staff needs) Group A: choose starting time and length of shift down to 15 minutes intervals. Group B: choose what days they wanted to work and not to work Group C: only choose between a few specific shifts. Duration: 12 months	Employees received training in the use of the software. All activities related to the interventions were financed by the organizations.	No intervention	Employee Need for recovery Sleep (disturbed sleep and awakening index) (Karolinska Scale), mental distress and somatic symptoms (reduced form of the Symptom Checklist-90) (Garde 2012)	Employee Work-life conflicts, work-life facilitation, marital conflicts, time with children (Albertsen 2014); influence on working hours, preferred length of duty/time of day/day to work, satisfaction with working hours (Garde 2012)	Employee Consider changing job (Garde 2012)
Almeida 2018—Supervisory/employee training (managing work-family interface)										
Almeida, et al. [23], McHale, et al. [31], Lawson, et al. [28], McHale, et al. [32], Davis, et al. [25], Kelly, et al. [27], Lee, et al. [29], Olson, et al. [33]	September 2009 to September 2011	USA	Cluster-randomized controlled trial (work units *n* = 56) Intervention-*n* = 27 involving 423 participants; control-29 involving 400 participants)	Employees of IT division of a large company (mean tenure was over 10 years) 823 employees completed interviews at baseline. 635 employees completed a weeklong actigraphy data collection (baseline). 147 employees with a child 9–17 years of age participated in an additional home interviews with their child. Median age: 46 ~60% male participants	STAR (Support-Transform-Achieve-Results) workplace intervention aimed at promoting employees’ schedule control and supervisor support for personal and family life Duration: employees 8h and managers attended an additional 4h Provider: outside facilitators Follow-up: up to 12 months	Sessions were held during work hours Four group facilitators delivered the STAR intervention to supervisors and employees (supported by research grants)	Usual practice	Employee Nighttime sleep duration, wake after sleep onset, daytime nap duration (Actigraphic sleep measures) (Lee 2016) Actigraphy-based total sleep duration and sleep quality, self-reported sleep insufficiency and insomnia symptoms (Olson 2015)	Employee Family Supportive Supervisor Behaviors (FSSB), schedule control, work-to-family conflict and family-to-work conflict, time adequacy with family, psychological job demands (Kelly 2014)	
								Employee/parent Cortisol awakening response (daily saliva collection) (Almeida 2018)	Employee/parent Time with children (Davis 2015)	
								Child Negative and positive affect (PANAS), daily stressful experiences (DISE) (Lawson 2016); sleep (PSQI; duration, variability, latency quality) (McHale 2015)	Child Parent-child relationships; parental warmth, parents’ education involvement, parents’ solicitation of information about youths’ daily experiences, time with parents (McHale 2016)	
Workplace parenting intervention (managing work-family interface)										
Haslam, et al. [26]	Not described	Australia	Randomized controlled trial (individual) Intervention *n* = 55; control *n* = 52	Teachers with at least one child between 2 and 12 years of age Mean age: 40.6 ~23.4% male participants	Workplace Triple P (WPTP, a workplace parenting intervention); aimed at reducing work–family conflict and improving work and family functioning in teachers (1) Three telephone consultations (2) The group section over two full days (9:00 am–4:30 pm including breaks) 1 week apart Duration: 3 weeks Provider: two registered psychologists Follow-up: 4 months	Two schools provided the intervention during paid work-time, the rest of the teachers attended outside school hours.	Waitlist control	Employee/parent Stress (Teacher Occupational Stress Factor Questionnaire, depression and anxiety (Depression-Anxiety-Stress Scale (DASS)	Employee/parent Work family conflict (Frone’s scale), job satisfaction, parental satisfaction (subscale of the Parenting Sense of Competence Scale), dysfunctional parenting, (Parenting scale), parental levels of self-efficacy (Parenting Task Checklist)	Employee/parent Teaching-related self-efficacy
								Child Problem behavior (Eyberg Child Behavior Inventory (ECBI)		
Martin and Sanders [30]	Not described	Australia	Randomized controlled trial (individual) Intervention *n* = 23; control *n* = 22	General and academic staff (employed for at least 20 hours per week) from a major metropolitan university with child aged between 2 and 9 years. Age range: 27 to 46 no data for gender	Workplace Triple P with 17 core positive parenting and child management strategies, and Planned Activities Training Duration: 8 weeks Provider: masters level psychologists (who were accredited Triple P providers) Follow-up: 4 months	WPTP delivered as part of a suite of evidence-based Employee Assistance Programs (EAP) funded by employers to promote ‘family friendly’ workplaces	Waitlist control	Employee/parent Parental adjustment (DASS), work stress	Employee/parent Dysfunctional parenting (Parenting scale), parenting efficacy (Problem Setting and Behavior Checklist), Social Support Scale, job satisfaction	Employee/parent Work commitment (Work Commitment Questionnaire), work-related self-efficacy
								Child Problem behavior (ECBI) (4-months) (The Strengths and Difficulties Questionnaire (SDQ)		
Sanders, et al. [34]	Not described	Australia	Randomized controlled trial (individual) Intervention *n* = 62 control *n* = 59	Employees employed at least half-time, with child aged between 1 and 16 years Various organizations (including State Government Departments of Education, Housing, Public Works, and Tourism, a large private hospital and a university) 27.6% fathers	Workplace Triple P consisted of two components: work-family balance coping skills and positive parenting skills Duration: 8 weeks period Provider: Triple P practitioners with postgraduate psychology qualifications Follow-up: up to 12 months	Group sessions were conducted at workplace during times identified as convenient by management and employees including lunchtimes, afternoons, or at the close of business	Waitlist control	Employee/parent Parental distress (DASS) (4-months), work stress (Work stress scale)	Employee/parent Dysfunctional parenting (Parenting scale), parenting efficacy (Parenting Task Checklist), job satisfaction	Employee/parent Work commitment (Cohen’s scale)
								Child Problem behavior (ECBI), child behavior (Strengths and Difficulties Questionnaire)		
Nunes 2017—Individualized counseling to employees (experiencing personal and work-related difficulties)										
Nunes, et al. [39], Richmond, et al. [40], Richmond, et al. [41]	October 2013 to July 2014	USA	Quasi-experimental design EAP users *n* =145 matched to non-EAP users *n* = 145 EAP users *n* = 156 matched to non-EAP users *n* = 188	Workers on EAP 19 departments of Colorado state government and the Judicial Branch No information on children Mean age: 44.6 ~30% male participants	Employee Assistance Programs (EAPs) offering individualized counseling to employees that support employees to identify effective coping strategies for personal and professional stressors Provider: Approximately 11 licensed staff members and 5–7 graduate student interns Follow-up: up to 12 months	The Colorado State Employee Assistance Program (C-SEAP) Intervention staff are employed by the state.	Non-EAP users	Employee Depression symptoms (PHQ), anxiety severity (GAD-2), alcohol use disorders (AUDIT), absenteeism, presenteeism, and workplace distress (Chestnut Global Partners Workplace Outcome Suite) (Richmond 2016)		Employee Sick time taken for a period of up to 12 months after the baseline survey (timecard data) (Nunes 2017); Absenteeism scale, presenteeism scale, workplace distress (Richmond 2017)

**Table 2:** Summary of intervention type, outcome measures ^†^, overall results and quality assessment of included studies.

Study id(s)	Intervention	Intervention Effect *	Primary Outcomes	Secondary Outcomes		Study Quality
			Health Outcomes	Social Wellbeing Outcomes	Job Performance Outcomes	
Schiller, et al. [35], Schiller, et al. [36], Barck-Holst, et al. [24]	Reduced working hour (cluster RCT)	Significant positive effect	Sleep quality Sleep duration (work day) Sleepiness Stress Worries/stress at bedtime	Work at workplace (work day) Domestic work (work day) (increase) Free time (both of work day and days off) (increase) Own time (work day) (increase) Time use patterns (workload decreased, recovery activities increased) Job demand, manager support, work intrusion on private life		Unclear risk of bias
		No significant difference	Sleep duration (days off) Daytime sleep time	Work from home Child care time/Care for others Personal care time Meal time Socializing time Rest time Daytime sleep		
Albertsen, et al. [37], Garde, et al. [38]	Self-rostering (flexibility) (non-RCT)	Significant positive effect	Need for recovery Disturbed sleep index Somatic symptoms Mental distress	Work–family facilitation Work–family conflict		High risk of bias
		Significant negative effect		Work–family facilitation, work–family conflict, time with children, satisfaction with working hours (one intervention arm) Consider changing job (one intervention arm)		
		No significant difference	Awakening index	Time with children Marital conflicts		
Almeida, et al. [23], McHale, et al. [31], Lawson, et al. [28], McHale, et al. [32], Davis, et al. [25], Kelly, et al. [27], Lee, et al. [29], Olson, et al. [33]	Supervisory/employee training (cluster RCT)	Significant positive effect	Total sleep time Sleep insufficiency Cortisol awakening response ^‡^ Sleep in child (Variability, Latency, Quality) ^‡^	Supervisor support for family/personal life ^‡^ Schedule control ^‡^ Family-to-Work conflict ^‡^ Enough time for family ^‡^ Daily parent-child time (no significant difference in fathers) ^‡^ Parent-child relationships (no significant effect in ITT analysis) ^‡^ Affective wellbeing in children ^§^		Low risk of bias
		No significant difference	Wake after sleep onset Insomnia symptoms Sleep in child (Duration)	Work-to-family conflict Psychological job demands		
Haslam, et al. [26], Martin and Sanders [30], Sanders, et al. [34]	Workplace parenting intervention (3 studies) (individual RCT)	Significant positive effect	Work stress ^‡^ Depression and anxiety ^‡^	Work–family conflict ^‡^ Parental satisfaction ^‡^ Dysfunctional parenting ^‡^ Parenting efficacy ^‡^ Work satisfaction ^‡^ Problem behavior (children) ^§^	Work efficacy ^‡^ Work commitment ^‡^	Unclear risk of bias
		No significant difference		Work satisfaction ^‡^ Problem behavior (children) ^§^		
Nunes, et al. [39], Richmond, et al. [40], Richmond, et al. [41]	Individualized counseling (non-RCT)	Significant positive effect	Depression symptoms Anxiety severity		Sick leave usage Presenteeism Absenteeism	Unclear risk of bias
		No significant difference	Workplace distress Alcohol Use Disorders			

^†^ Outcome measures refer to the review primary and secondary outcomes, not those for individual studies; * Describes intervention effect in entire study population with or without statistical significance at *p* < 0.05 level (details clinical significance in terms of effect sizes is provided in Supplementary Material S5); ^‡^ Shows outcome in employee/parents and ^§^ indicates outcome in children. All other results refer to general employee outcome.

## Data Availability

Not applicable.

## Supplementary Materials

The following supporting information can be downloaded at: https://www.mdpi.com/article/10.3390/ijerph19084779/s1, Supplementary Material S1: PRISMA Checklists; Supplementary Material S2: Search strategies; Supplementary Material S3: template data collection forms; Supplementary Material S4: List of excluded reviews with reasons; Supplementary Material S5: Description of intervention, analyses, outcomes assessed and results of included studies.

## Appendix A

The review PICO and Risk of Bias tables assessed using the Cochrane Collaboration’s tool for assessing risk of bias for included RCT studies and the Risk of Bias Assessment Tool for Non-Randomized Studies (RoBANS) for non-RCT included studies.

**Table A1:** Study PICO criteria.

Population	
Employed fathers or parents (including adults of child rearing age, working in full- or part-time capacity regardless of if they are parents or not)	
Intervention	Comparator
Workplace interventions focused on work flexibility (time schedule, place), work demands (working hour, workload), and leave and days off (paternity leave, childcare leave)	No intervention, other active arms, placebo, wait list, and usual practice
Critical Outcomes	
Physical health (e.g., fatigue, sickness), mental health (e.g., depression, anxiety, stress), and general health (e.g., sleepiness) in fathers, mothers, and children Social wellbeing, such as: ○ quality of life (QOL) ○ work-life balance (including time spent with children) ○ couple and parent-child relationship ○ social support Job performance, such as: ○ absenteeism and ○ presenteeism	

**Table A2:** Risk of Bias for included RCT studies.

	Almeida 2018	Haslam 2013	Martin 2003	Sanders 2011	Schiller 2017
Random sequence generation	Low	Low	Unclear	Low	Unclear
Allocation concealment	Low	Low	Unclear	Low	Unclear
Blinding of participants and personnel	Unclear (insufficient information)	High	High (no information)	High (no information)	Unclear (insufficient information)
Blinding of outcome assessment	Unclear (training/self-reported)	Unclear (training/self-reported)	Unclear (training/self-reported)	Unclear (training/self-reported)	Unclear (Reduced working hour)
Incomplete outcome data	Low	Low	High	Low	Low
Selective reporting	Unclear	Unclear	Unclear	Unclear	Unclear
Other bias	Low	Low	Low	Low	Low
Summary assessment	Low risk of bias	Unclear risk of bias	Unclear risk of bias	Unclear risk of bias	Unclear risk of bias

**Table A3:** Risk of Bias for non-RCT included studies.

Non RCTs	Albertsen 2014	Nunes 2017
Selection of participants	High	Low
Confounding variables	Low	Low
Measurement of exposure	Unclear	High
Blinding of outcome assessment	Unclear	Unclear
Incomplete outcome data	Low	Unclear
Selective outcome reporting	Unclear	Unclear
Summary assessment	High risk of bias	Unclear risk of bias
