# Supplementary material for: Effectiveness of Workplace Interventions for Improving Working Conditions on the Health and Wellbeing of Fathers or Parents: A Systematic Review
Source: Int J Environ Res Public Health. 2022 Apr 14;19(8):4779. doi: 10.3390/ijerph19084779 (PMC9027029; doi:10.3390/ijerph19084779)
Supplement: Supplementary file 1 [file ijerph-19-04779-s001.zip › Suto et.al_supplementary_material_S2.pdf]

Supplementary material 2: Search strategies

Medline (1946 to February 26, 2020)

#1	Parents/ or Fathers/ or Single parent/ or Spouses/ or (father* or parent* or partner* or spouse*).mp.	677673
#2	exp Family Leave/	977
#3	((parental or paternity or paternal or family or childcar* or "child car*") adj leave).mp.	1225
#4	(career* adj1 break*).mp.	72
#5	exp "Personnel Staffing and Scheduling"/	40156
#6	("work life balance" or "worklife balance" or "work to family" or "work family balance").mp.	3728
#7	(flexitime* or "flex time*" or overtime or shiftwork* or nightwork* or telework* or daywork* or "job* shar*" or "decent work*" or presenteeism or absenteeism).mp.	16094
#8	(work* adj1 (schedul* or day*1 or hour*1 or flexible or place flexibility or time*1 or home or remot* or distributed or arrangement* or (life adj2 polic*))).mp.	30218
#9	(commut* adj3 (time or hour*)).mp.	394
#10	((staffing or control) adj1 schedul*).mp.	297
#11	((contingent or atypical) adj employ*).mp.	19
#12	(((official or business) adj1 trip) or (travel adj1 business)).mp.	92
#13	(((day*1 or night*1 or late or early or evening or core or stagger* or compress* or irregular) adj2 (work* or shift*1 or hour*1)) or overtime).mp.	40650
#14	((annual* or core or reduc* or stagger* or compress*) adj2 hour*1).mp.	2714

#15	("triple p" or "family friendly" or "work family health network" or "support transform achieve results").mp.	537
#16	or/2-15	107785
#17	1 and 16	6154
#18	Work/ or Workplace/ or Employment/	84560
#19	((work* or job* or employment*) adj1 (place* or site* or location* or setting* or office*)).mp.	11963
#20	(factory or factories or company or companies or employee* or employer* or "human resource*" or business* or employee* or employer* or "small and medium enterprises" or SMEs or worker* or workplace* or worksite* or staff or personnel).mp.	811822
#21	or/18-20	857263
#22	17 and 21	2232
#23	exp animals/ not humans.sh.	4672688
**#24**	**22 not 23**	**2230**

1.1 EMBASE (29 Feb 2020)

#1	('parent'/de OR 'father'/exp OR 'single parent'/de OR 'spouse'/exp OR father*:ab,ti OR parent*:ab,ti OR partner*:ab,ti OR spouse*:ab,ti) AND [embase]/lim NOT [medline]/lim	237529
#2	('work-life balance'/de OR 'work schedule'/de OR 'working time'/de OR 'shift work'/exp OR 'telecommuting'/de OR 'absenteeism'/de OR 'presenteeism'/de) AND [embase]/lim NOT [medline]/lim	12039
#3	'family leave'/exp AND [embase]/lim NOT [medline]/lim	83
#4	(((parental OR paternity OR paternal OR family OR childcar* OR 'child car*') NEXT/1 leave):ab,ti) AND [embase]/lim NOT [medline]/lim	84

#5	((career* NEAR/1 break*):ab,ti) AND [embase]/lim NOT [medline]/lim	18
#6	'personnel management'/exp AND [embase]/lim NOT [medline]/lim	11956
#7	('work-life balance':ab,ti OR 'work life balance':ab,ti OR 'worklife balance':ab,ti OR 'work to family':ab,ti OR 'work family balance':ab,ti) AND [embase]/lim NOT [medline]/lim	601
#8	(flexitime*:ab,ti OR 'flex time*':ab,ti OR overtime:ab,ti OR shiftwork*:ab,ti OR nightwork*:ab,ti OR telework*:ab,ti OR daywork*:ab,ti OR 'job* shar*':ab,ti OR 'decent work*':ab,ti OR presenteeism:ab,ti OR absenteeism:ab,ti) AND [embase]/lim NOT [medline]/lim	6901
#9	(((work* NEAR/1 (schedul* OR day*1 OR hour*1 OR flexible OR 'place flexibility' OR time*1 OR home OR remot* OR distributed OR arrangement*)):ab,ti) OR ((life NEAR/2 polic*):ab,ti)) AND [embase]/lim NOT [medline]/lim	1967
#10	((commut* NEAR/3 (time OR hour*)):ab,ti) AND [embase]/lim NOT [medline]/lim	178
#11	(((staffing OR control) NEAR/1 schedul*):ab,ti) AND [embase]/lim NOT [medline]/lim	145
#12	(((contingent OR atypical) NEXT/1 employ*):ab,ti) AND [embase]/lim NOT [medline]/lim	5
#13	((((official OR business) NEAR/1 trip):ab,ti) OR ((travel NEAR/1 business):ab,ti)) AND [embase]/lim NOT [medline]/lim	23
#14	((((day OR days OR night OR nights OR late OR early OR evening OR core OR stagger* OR compress* OR irregular) NEAR/2 (work* OR shift*1 OR hour*1)):ab,ti) OR overtime:ab,ti) AND [embase]/lim NOT [medline]/lim	11425
#15	(((annual* OR core OR reduc* OR stagger* OR compress*) NEAR/2 hour*1):ab,ti) AND [embase]/lim NOT [medline]/lim	0
#16	('triple p':ab,ti OR 'family friendly':ab,ti OR 'work family health network':ab,ti OR 'support transform achieve results':ab,ti) AND [embase]/lim NOT [medline]/lim	223
#17	#2 OR #3 OR #4 OR #5 OR #6 OR #7 OR #8 OR #9 OR #10 OR #11 OR #12 OR #13 OR #14 OR #15 OR #16	35278

#18	#1 AND #17	1936
#19	'workplace'/exp AND [embase]/lim NOT [medline]/lim	12691
#20	(((work* OR job* OR employment*) NEAR/1 (place* OR site* OR location* OR setting* OR office*)):ab,ti) AND [embase]/lim NOT [medline]/lim	5277
#21	(factory:ab,ti OR factories:ab,ti OR company:ab,ti OR companies:ab,ti OR 'human resource*':ab,ti OR business*:ab,ti OR employee*:ab,ti OR employer*:ab,ti OR 'small and medium enterprise*':ab,ti OR smes:ab,ti OR worker*:ab,ti OR workplace*:ab,ti OR worksite*:ab,ti OR staff:ab,ti OR staffs:ab,ti OR personnel:ab,ti) AND [embase]/lim NOT [medline]/lim	248100
#22	#19 OR #20 OR #21	252433
#23	#18 AND #22	606
**#24**	**#23 NOT ([animals]/lim NOT [humans]/lim)**	**601**

1.2 CENTRAL (02 Feb 2020)

#1	MeSH descriptor: [Parents] this term only	2919
#2	MeSH descriptor: [Fathers] this term only	169
#3	MeSH descriptor: [Single Parent] this term only	44
#4	MeSH descriptor: [Spouses] this term only	339
#5	((father* OR parent* OR partner* OR spouse*)) (Word variations have been searched)	59613
#6	#1 OR #2 OR #3 OR #4 #5	3449
#7	MeSH descriptor: [Family Leave] explode all trees	4
#8	(parental OR paternity OR paternal OR family OR childcar* OR "child car*") NEXT leave	13

#9	career* NEAR/1 break*	1
#10	MeSH descriptor: [Personnel Staffing and Scheduling] explode all trees	579
#11	work life balance OR "worklife balance" OR "work to family" OR "work family balance"	49
#12	flexitime* OR "flex time*" OR overtime OR shiftwork* OR nightwork* OR telework* OR daywork* OR "job* shar*" OR "decent work*" OR presenteeism OR absenteeism	2455
#13	work* NEAR/1 (schedul* OR day OR days OR hour OR hours OR flexible OR “place flexibility” OR time OR times OR home OR remot* OR distributed OR arrangement* OR (life NEAR/2 polic*))	3626
#14	commut* NEAR/3 (time or hour*)	37
#15	(staffing OR control) NEAR/1 schedul*	84
#16	(contingent OR atypical) NEXT employ*	4
#17	((official OR business) NEAR/1 trip) OR (travel NEAR/1 business)	5
#18	((day OR days OR night OR nights OR late OR early OR evening OR core OR stagger* OR compress* OR irregular) NEAR/2 (work* OR shift OR shifts OR hour OR hours)) OR overtime	9933
#19	(annual* OR core OR reduc* OR stagger* OR compress*) NEAR/2 (hour OR hours)	976
#20	triple p OR "family friendly" OR "work family health network" OR "support transform achieve results"	255
#21	#7 OR #8 OR #9 OR #10 OR #11 OR #12 OR #13 OR #14 OR #15 OR #16 OR #17 OR #18 OR #19 OR #20	15249
#22	#6 AND #21	100
#23	MeSH descriptor: [Work] this term only	219
#24	MeSH descriptor: [Workplace] this term only	808

#25	MeSH descriptor: [Employment] this term only	703
#26	(work* OR job* OR employment*) NEAR/1 (place* OR site* OR location* OR setting* OR office*)	1497
#27	(factory OR factories OR company OR companies OR employee* OR employer* OR "human resource*" OR business* OR employee* OR employer* OR "small and medium enterprise" OR "small and medium enterprises" OR smes OR worker* OR workplace* OR worksite* OR staff OR staffs OR personnel)	57586
#28	#23 OR #24 OR #25 OR #26 OR #27	58554
#29	#22 AND #28	22
#30	#22 AND #28 in Cochrane Reviews	11
**#31**	**#22 AND #28 in Trials**	**11**

1.3 PsycINFO (1806 to February Week 4 2020)

#1	Parents/ or Single Parents/ or exp Fathers/ or Expectant parents/ or Expectant fathers/ or Spouses/ or Husbands/ or Partners/ or (parent* or father* or spouse* or husband* or partner*).mp.	429497
#2	Employee leave benefits/	1070
#3	((parental or paternity or paternal or family or childcar* or "child car*") adj leave).mp.	573
#4	(career* adj1 break*).mp.	46
#5	exp Work scheduling/ or Work rest cycles/ or Work-life balance/	2657
#6	("work life balance" or "worklife balance" or "work to family").mp.	8574
#7	(flexitime* or "flex time*" or overtime or shiftwork* or nightwork* or telework* or daywork* or "job* shar*" or "decent work*" or presenteeism or absenteeism).mp.	7818

#8	(work* adj1 (schedul* or day*1 or hour*1 or flexible or place flexibility or time*1 or home or remot* or distributed or arrangement* or (life adj2 polic*))).mp.	14131
#9	(commut* adj3 (time or hour*)).mp.	196
#10	((staffing or control) adj1 schedul*).mp.	426
#11	((contingent or atypical) adj employ*).mp.	84
#12	(((official or business) adj1 trip) or (travel adj1 business)).mp.	94
#13	(((day*1 or night*1 or late or early or evening or core or stagger* or compress* or irregular) adj2 (work* or shift*1 or hour*1)) or overtime).mp.	13280
#14	((annual* or core or reduc* or stagger* or compress*) adj2 hour*1).mp.	475
#15	("triple p" or "family friendly" or "work family health network" or "support transform achieve results").mp.	992
#16	or/2-15	39092
#17	1 and 16	6592
#18	Workplace intervention/ or exp Employment status/	26704
#19	((work* or job* or employment*) adj1 (place* or site* or location* or setting* or office*)).mp.	10380
#20	(factory or factories or company or companies or employee* or employer* or "human resource*" or business* or employee* or employer* or "small and medium enterprises" or SMEs or worker* or workplace* or worksite* or staff or personnel).mp.	472050
#21	or/18-20	490509
**#22**	**17 and 21**	**2729**

1.4 ERIC (08 March 2020)

S1	(Ti,ab(father* OR parent* OR partner* OR spouse*) OR MAINSUBJECT.EXACT.EXPLODE("Parents") OR MAINSUBJECT.EXACT.EXPLODE("Fathers") OR MAINSUBJECT.EXACT.EXPLODE("One Parent Family") OR MAINSUBJECT.EXACT.EXPLODE("Spouses"))	167980
S2	Ti,ab(flexitime* OR "flex time" OR overtime OR shiftwork* OR nightwork* OR telework* OR daywork* OR "job* shar*" OR ("decent work" OR "decent working") OR presenteeism OR absenteeism) OR ((Ti,ab(family PRE/0 leave) OR Ti,ab((parental OR paternity OR paternal OR family OR childcar*) PRE/0 leave)) OR (Ti,ab(career* PRE/1 break*) OR Ti,ab(Personnel PRE/0 Staffing PRE/1 Scheduling*)) OR Ti,ab("work life balance" OR "worklife balance" OR "work to family" OR "work family balance")) OR (Ti,ab(commut* PRE/3 (time OR hour*)) OR Ti,ab((staffing OR control) PRE/1 schedul*) OR Ti,ab((contingent OR atypical) PRE/0 employ*) OR Ti,ab((official OR business) PRE/1 trip OR travel PRE/1 business)) OR Ti,ab(work* PRE/1 (schedul* OR day? OR hour? OR flexible OR "place flexibility" OR time? OR home OR remot* OR distributed OR arrangement* OR life PRE/2 polic*)) OR (Ti,ab(((day OR days OR night OR nights OR late OR early OR evening OR core OR stagger* OR compress* OR irregular) PRE/2 (work* OR shift* OR hour*)) OR overtime) OR Ti,ab((annual* OR core OR reduc* OR stagger* OR compress*) PRE/2 hour*) OR Ti,ab("triple p" OR "family friendly" OR "work family health network" OR "pport transform achieve results"))	12163
S3	((MAINSUBJECT.EXACT.EXPLODE("Workplace Literacy") OR MAINSUBJECT.EXACT.EXPLODE("Workplace Learning") OR MAINSUBJECT.EXACT.EXPLODE("Employment")) OR Ti,ab((work* OR job* OR employment*) PRE/1 (place* OR site* OR location* OR setting* OR office*)) OR Ti,ab(factory OR factories OR company OR companies OR employee* OR employer* OR "human resource" OR "human resources" OR business* OR employee* OR employer* OR "small and medium enterprise*" OR smes OR worker* OR workplace* OR worksite* OR staff OR staffs OR personnel))	226315
S4	(Ti,ab(father* OR parent* OR partner* OR spouse*) OR MAINSUBJECT.EXACT.EXPLODE("Parents") OR MAINSUBJECT.EXACT.EXPLODE("Fathers") OR MAINSUBJECT.EXACT.EXPLODE("One Parent Family") OR MAINSUBJECT.EXACT.EXPLODE("Spouses")) AND ((MAINSUBJECT.EXACT.EXPLODE("Workplace Literacy") OR MAINSUBJECT.EXACT.EXPLODE("Workplace Learning") OR MAINSUBJECT.EXACT.EXPLODE("Employment")) OR Ti,ab((work* OR job* OR employment*) PRE/1 (place* OR site* OR location* OR setting* OR office*)) OR Ti,ab(factory OR factories OR company OR companies OR employee* OR employer* OR "human resource" OR "human resources" OR business* OR employee* OR employer* OR "small and medium enterprise*" OR	**1113**

	smes OR worker* OR workplace* OR worksite* OR staff OR staffs OR personnel)) AND (Ti,ab(flexitime* OR "flex time" OR overtime OR shiftwork* OR nightwork* OR telework* OR daywork* OR "job* shar*" OR ("decent work" OR "decent working") OR presenteeism OR absenteeism) OR ((Ti,ab(family PRE/0 leave) OR Ti,ab((parental OR paternity OR paternal OR family OR childcar*) PRE/0 leave)) OR (Ti,ab(career* PRE/1 break*) OR Ti,ab(Personnel PRE/0 Staffing PRE/1 Scheduling*)) OR Ti,ab("work life balance" OR "worklife balance" OR "work to family" OR "work family balance")) OR (Ti,ab(commut* PRE/3 (time OR hour*)) OR Ti,ab((staffing OR control) PRE/1 schedul*) OR Ti,ab((contingent OR atypical) PRE/0 employ*) OR Ti,ab((official OR business) PRE/1 trip OR travel PRE/1 business)) OR Ti,ab(work* PRE/1 (schedul* OR day? OR hour? OR flexible OR "place flexibility" OR time? OR home OR remot* OR distributed OR arrangement* OR life PRE/2 polic*)) OR (Ti,ab(((day OR days OR night OR nights OR late OR early OR evening OR core OR stagger* OR compress* OR irregular) PRE/2 (work* OR shift* OR hour*)) OR overtime) OR Ti,ab((annual* OR core OR reduc* OR stagger* OR compress*) PRE/2 hour*) OR Ti,ab("triple p" OR "family friendly" OR "work family health network" OR "pport transform achieve results")))	

1.5 SSCI (3 April 2020)

# 1	TS=(father* OR parent* OR partner* OR spouse*)	282,036
# 2	TS="One Parent Family"	28
# 3	TS="Spouses"	7,251
# 4	#3 OR #2 OR #1	282,036
# 5	TS=”family leave”	184
# 6	Ts=((parental OR paternity OR paternal OR family OR childcar*) NEAR/0 leave)	1,046
# 7	ts=((career* NEAR/1 break*) OR “Personnel Staffing and Scheduling*” OR "work life balance" OR "worklife balance" OR "work to family" OR "work family balance")	2,777
# 8	ts=(flexitime* OR "flex time*" OR overtime OR shiftwork* OR nightwork* OR telework* OR daywork* OR "job* shar*" OR ("decent work" OR "decent working") OR presenteeism OR absenteeism)	7,609

# 9	ts=(work* NEAR/1 (schedul* OR day? OR hour? OR flexible OR "place flexibility" OR time? OR home OR remot* OR distributed OR arrangement* OR (life NEAR/2 polic*)))	14,815
# 10	ts=(commut* NEAR/3 (time OR hour*) OR (staffing OR control) NEAR/1 schedul* OR (contingent OR atypical) NEAR/0 employ*)	1,580
# 11	ts=((official OR business) NEAR/1 trip OR (travel NEAR/1 business))	328
# 12	ts=(((day OR days OR night OR nights OR late OR early OR evening OR core OR stagger* OR compress* OR irregular) NEAR/2 (work* OR shift* OR hour*)) OR overtime)	21,040
# 13	ts=((annual* or core or reduc* or stagger* or compress*) NEAR/2 hour*)	1,049
# 14	ts=("triple p" or "family friendly" or "work family health network" or "pport transform achieve results")	1,058
# 15	#14 OR #13 OR #12 OR #11 OR #10 OR #9 OR #8 OR #7 OR #6 OR #5	43,046
# 16	ts=((work* OR job* OR employment*) NEAR/1 (place* OR site* OR location* OR setting* OR office*))	10,106
# 17	ts=(factory OR factories OR company OR companies OR employee* OR employer* OR "human resource" OR "human resources" OR business* OR employee* OR employer* OR "small and medium enterprise*" OR smes OR worker* OR workplace* OR worksite* OR staff OR staffs OR personnel)	409,634
# 18	#17 OR #16	413,782
**# 19**	**#18 AND #15 AND #4**	**1,545**
