# Supplementary material for: Effectiveness of Workplace Interventions for Improving Working Conditions on the Health and Wellbeing of Fathers or Parents: A Systematic Review
Source: Int J Environ Res Public Health. 2022 Apr 14;19(8):4779. doi: 10.3390/ijerph19084779 (PMC9027029; doi:10.3390/ijerph19084779)
Supplement: Supplementary file 1 [file ijerph-19-04779-s001.zip › Suto et.al_supplementary_material_S3.pdf]

Supplementary material 3: template data collection forms
Data collection form– RCTs and NRS

Review title or ID	
Study ID *(Study List: column C)*	
Report ID *(Study List: column D)*	
Name of person extracting data	

Characteristics of included studies

Methods

	**Descriptions as stated in report/paper**	**Location in text or source** *(pg & ¶/fig/table/other)*
**Aim of study** *(e.g. efficacy, equivalence, pragmatic)*		
**Design** *(e.g. parallel, crossover, quasi, before and after, interrupted time series)*		
**Unit of allocation** *(by individuals, cluster/ groups or body parts)*		
**Start date**		
**End date**		
**Notes:**		

Participants

	Description<br>*Include comparative information for each intervention or comparison group if available*	Location in text or source *(pg & ¶/fig/table/other)*
Population description *(from which study participants are drawn)*		
Setting *(including type of workplace/industry)*		
Inclusion criteria		
Exclusion criteria		
Method of recruitment of participants *(e.g. phone, mail, clinic patients)*		
Total no. randomised *(or total pop. at start of study for NRCTs)*		
Clusters *(if applicable, no., type, no. people per cluster)*		
Withdrawals and exclusions *(if not provided below by outcome)*		
Age		
Age of children		
Sex		
Race/Ethnicity		
Other relevant sociodemographics		
Notes:		

Intervention groups

Copy and paste table for each intervention and comparison group

Intervention Group 1

	Description as stated in report/paper	Location in text or source *(pg & ¶/fig/table/other)*
Group name		
No. randomised to each group *(specify whether no. people or clusters)*		
Theoretical basis *(include key references)*		
Description *(include targeted working condition: flexibility, work demands, leave and days off)*		
Duration of treatment period		
Frequency of treatment period		
Delivery mode *(e.g. individual, group, online, telephone)*		
Providers *(e.g. no., profession, training, ethnicity etc. if relevant)*		
Economic information *(i.e. intervention cost, changes in other costs as result of intervention)*		
Resource requirements *(e.g. staff numbers, cold chain, equipment)*		
Comparison Description *(e.g. as usual, other active arm , no intervention, wait-list)*		
Notes:		

Outcomes

Copy and paste table for each outcome.

Outcome 1

	Description as stated in report/paper		Location in text or source *(pg & ¶/fig/table/other)*
Outcome name			
Type of outcomes *(physical health, mental health, general health, social wellbeing, job performance)*			
Time points reported			
Outcome definition *(with diagnostic criteria if relevant)*			
Person measuring/ reporting			
Is outcome/tool validated?	☐ Yes ☐ No ☐ Unclear		
Imputation of missing data *(e.g. assumptions made for ITT analysis)*			
Assumed risk estimate *(e.g. baseline or population risk noted in Background)*			
Notes:			

Other

**Study funding sources** *(including role of funders)*		
**Possible conflicts of interest** *(for study authors)*		
**Project management**		

Risk of Bias assessment

For RCTs

(See Chapter 8 of the Cochrane Handbook.)

Domain	Risk of bias<br>Low High Unclear	Support for judgement<br>*(include direct quotes where available with explanatory comments)*	Location in text or source *(pg & ¶/fig/table/other)*
Random sequence generation *(selection bias)*	☐ ☐ ☐		
Allocation concealment *(selection bias)*	☐ ☐ ☐		
Blinding of participants and personnel *(performance bias)*	☐ ☐ ☐	Outcome group: All/	
(if separate judgement by outcome(s) required)	☐ ☐ ☐	Outcome group:	
Blinding of outcome assessment *(detection bias)*	☐ ☐ ☐	Outcome group: All/	
(if separate judgement by outcome(s) required)	☐ ☐ ☐	Outcome group:	
Incomplete outcome data *(attrition bias)*	☐ ☐ ☐	Outcome group: All/	
(if separate judgement by outcome(s) required)	☐ ☐ ☐	Outcome group:	
Selective outcome reporting? *(reporting bias)*	☐ ☐ ☐		
Other bias	☐ ☐ ☐		
Notes:			

For non-RCTs

(See ROBANS file.)

Domain	Risk of bias<br>Low High Unclear	Support for judgement<br>*(include direct quotes where available with explanatory comments)*	Location in text or source *(pg & ¶/fig/table/other)*
The selection of participants *(selection bias)*	☐ ☐ ☐		
Confounding variables *(selection bias)*	☐ ☐ ☐		
Measurement of exposure *(performance bias)*	☐ ☐ ☐	Outcome group: All/	
(if separate judgement by outcome(s) required)	☐ ☐ ☐	Outcome group:	
Blinding of outcome assessment *(detection bias)*	☐ ☐ ☐	Outcome group: All/	
(if separate judgement by outcome(s) required)	☐ ☐ ☐	Outcome group:	
Incomplete outcome data *(attrition bias)*	☐ ☐ ☐	Outcome group: All/	
(if separate judgement by outcome(s) required)	☐ ☐ ☐	Outcome group:	
Selective outcome reporting? *(reporting bias)*	☐ ☐ ☐		
Other bias	☐ ☐ ☐		
Notes:			

Data and analysis

- The primary outcome measures include physical health (e.g. fatigue, sickness), mental health (e.g. depression, anxiety, stress), and general health (e.g. sleepiness) in fathers, mothers, and children.
- The secondary outcome measures include social wellbeing, such as quality of life (QOL), work life balance (including time spent with children), couple and parent-child relationship, and social support. Job performance including absenteeism and presenteeism will be also included.

Copy and paste the appropriate table for each outcome, including additional tables for each time point and subgroup as required.

Dichotomous outcome

	Description as stated in report/paper				Location in text or source *(pg & ¶/fig/table/other)*
Comparison					
Outcome					
Subgroup					
Time point *(specify from start or end of intervention)*					
Results	Intervention		Comparison		
	No. with event	Total in group	No. with event	Total in group	
Any other results reported *(e.g. odds ratio, risk difference, CI or P value)*					
No. missing participants					
Reasons missing					
Notes:					

Continuous outcome

	Description as stated in report/paper	Location in text or source *(pg & ¶/fig/table/other)*
Comparison		
Outcome		
Subgroup		

Time point *(specify from start or end of intervention)*							
Results	Intervention			Comparison			
	Mean	SD *(or other variance, specify)*	No. participants	Mean	SD *(or other variance, specify)*	No. participants	
Any other results reported *(e.g. mean difference, CI, P value)*							
No. missing participants							
Reasons missing							
Notes:							

Other information

	Description as stated in report/paper	Location in text or source *(pg & ¶/fig/table/other)*
**Key conclusions of study authors**		
**Trial registration** *(URL)*		
**Notes:**		

Sources:

Cochrane Collaboration Glossary, 2010. Available from http://www.cochrane.org/training/cochrane-handbook.

Higgins JPT, Green S (editors). Cochrane Handbook for Systematic Reviews of Interventions Version 5.1.0 [updated March 2011]. The Cochrane Collaboration, 2011. Available from www.cochrane-handbook.org.

Last JM (editor), A Dictionary of Epidemiology, 4th Ed. New York: Oxford University Press, 2001.

Schünemann H, Brożek J, Oxman A, editors. GRADE handbook for grading quality of evidence and strength of recommendation. Version 3.2 [updated March 2009]. The GRADE Working Group, 2009. Available from http://www.cc-ims.net/gradepro.
