# Supplementary material for: Effectiveness of Workplace Interventions for Improving Working Conditions on the Health and Wellbeing of Fathers or Parents: A Systematic Review
Source: Int J Environ Res Public Health. 2022 Apr 14;19(8):4779. doi: 10.3390/ijerph19084779 (PMC9027029; doi:10.3390/ijerph19084779)
Supplement: Supplementary file 1 [file ijerph-19-04779-s001.zip › Suto et.al_supplementary_material_S4.pdf]

Supplementary material 4: List of excluded reviews with reasons

	Study	Reason for exclusion
1.	Baugh E, Ballard SM, Tyndall L, Littlewood K, Nolan M: Balancing work and family: A pilot evaluation of an evidence-based parenting education program. Families in Society 2015, 96(3):195-202.	Study design: not Controlled trial
2.	Bocchicchio AM: Fathers in the workplace: the use of EAP core technology functions in assisting fathers with work/family balance. Journal of Workplace Behavioral Health 2007, 22(1):89-102.	Study design: review article
3.	Brady JM: Family Linked Workplace Resources and Contextual Factors as Important Predictors of Job and Individual Well-being for Employees and Families. 2019.	Publication type: thesis
4.	Dikkers J, Den Dulk L, Geurts S, Peper B: Work-nonwork culture, utilization of work-nonwork arrangements, and employee-related outcomes in two Dutch organizations. Work and family: An international research perspective 2005:118-139.	Publication type: book
5.	Duxbury LE, Higgins CA, Thomas DR: Work and family environments and the adoption of computer-supported supplemental work-at-home. Journal of Vocational Behavior 1996, 49(1):1-23.	Study design: not Controlled trial
6.	Gartzia L, Sánchez-Vidal ME, Cegarra-Leiva D: Male leaders with paternity leaves: effects of work norms on effectiveness evaluations. European Journal of Work and Organizational Psychology 2018, 27(6):793-808.	Study design: not Controlled trial
7.	Gjerdingen DK, Center B: A randomized controlled trial testing the impact of a support/work-planning intervention on first-time parents' health, partner relationship, and work responsibilities. Behavioral Medicine 2002, 28(3):84-91.	Intervention: not workplace intervention
8.	Hartung D, Hahlweg K: Strengthening parent well-being at the work—family interface: A German trial on workplace Triple P. Journal of community & applied social psychology 2010, 20(5):404-418.	Intervention: not workplace intervention
9.	Hartung D, Hahlweg K: Stress reduction at the work-family interface: Positive parenting and self-efficacy as mechanisms of change in Workplace Triple P. Behavior modification 2011, 35(1):54-77.	Intervention: not workplace intervention
10.	Matias M, Ferreira T, Vieira J, Cadima J, Leal T, Mena Matos P: Workplace family support, parental satisfaction, and work–family conflict: Individual and	Study design: not Controlled trial

crossover effects among dual-earner couples. Applied Psychology 2017, 66(4):628-652.	
11. McLoyd VC, Kaplan R, Purtell KM, Huston AC: Assessing the effects of a work-based antipoverty program for parents on youth’s future orientation and employment experiences. Child Development 2011, 82(1):113-132.	Intervention: not interventions for working conditions
12. Michaud TL, Nyman JA, Jutkowitz E, Su D, Dowd B, Abraham JM: Effect of workplace weight management on health care expenditures and quality of life. Journal of occupational and environmental medicine 2016, 58(11):1073-1078.	Intervention: not interventions for working conditions
13. Secret M: Parenting in the workplace: Child care options for consideration. The Journal of Applied Behavioral Science 2005, 41(3):326-347.	Intervention: not interventions for working conditions
