# Supplementary material for: Effectiveness of Workplace Interventions for Improving Working Conditions on the Health and Wellbeing of Fathers or Parents: A Systematic Review
Source: Int J Environ Res Public Health. 2022 Apr 14;19(8):4779. doi: 10.3390/ijerph19084779 (PMC9027029; doi:10.3390/ijerph19084779)
Supplement: Supplementary file 1 [file ijerph-19-04779-s001.zip › Suto et.al_supplementary_material_S5.pdf]

Supplementary material 5: Description of intervention, analyses, outcomes assessed and results of included studies

**Table S3**: Description of intervention, analyses, outcomes assessed and results of included studies

| Study ID | Intervention (Number of participants) | Comparator | Analyses | Outcome (assessment period) | Results (95% Confidence interval) | Comments |
|---|---|---|---|---|---|---|
| Reduced weekly working hours | | | | | | |
| Schiller, et al. (2017) [35], Schiller, et al. (2018) [36], Barck-Holst, et al. (2017) [24] | Reduced weekly working hours by 25% (n = 354) (N = 17)<br>Men: about 25% (subgroup analysis by gender)<br>Fathers: N/A | Control group: no intervention (n = 226) (N = 16) | (Schiller 2017)<br>・Multilevel mixed model<br>・Model included "group" (intervention versus control), "time" (baseline, 9 and 18 months) and the group × time interaction.<br>・Subgroup analyses: gender, age, having children living at home, and baseline levels of sleep quality and worries and stress at bedtime.<br>*Main effect: group × time interaction, all three data collection periods (baseline, 9 and 18 months) are included in the model.<br>*Estimate: Coefficient | Employee (male and female) | | "The hypothesis that a 25% worktime reduction from fulltime work during 18 months would result in longer sleep duration on workdays, better subjective sleep quality, lower mean daytime sleepiness and perceived stress, including worries and stress at bedtime, was confirmed."<br><br>Subgroup analysis: Neither women, participants with children living at home, nor older employees did benefit more from worktime reduction." |
| | | | | SSQ (sleep quality, 1–5 good) (18 months) | **Work day: Estimate 0.086 (0.036 to 0.135) (P < 0.01)** | |
| | | | | | **Days off: Estimate 0.109 (0.040 to 0.179) (P < 0.01)** | |
| | | | | Sleep duration (hh:min) (18 months) | **Work day: Estimate 0.196 (0.130 to 0.263) (P < 0.01)** | |
| | | | | | Days off: Estimate -0.042 (-0.159 to 0.074) | |
| | | | | Sleepiness (1–9 very sleepy) (18 months) | **Work day: Estimate -0.206 (-0.295 to -0.117) (P < 0.01)** | |
| | | | | | **Days off: Estimate: -0.224 (-0.337 to -0.111) (P < 0.01)** | |
| | | | | Stress (1–9 very high) (18 months) | **Work day: Estimate: -0.243 (-0.356 to -0.130) (P < 0.01)** | |
| | | | | | **Days off: Estimate: -0.224 (-0.338 to -0.110) (P < 0.01)** | |
| | | | | Worries/stress at bedtime (1–5 no worry/stress) (18 months) | **Work day: Estimate: 0.089 (0.026 to 0.151) (P < 0.01)** | |
| | | | | | **Days off: Estimate: 0.098 (0.032 to 0.164) (P < 0.01)** | |
| | | | (Schiller 2018)<br>・Multilevel mixed model<br>・Model included "group" (intervention versus control), "time" (baseline, 9 and 18 months) and the group × time interaction.<br>・Subgroup analyses: gender, living with a partner, living alone with children, working are, and working shift.<br>*Main effect: group × time interaction, all three data collection periods (baseline, 9 and 18 months) are included in the model.<br>*Alpha level of 0.01 was used. | Work at workplace (18 months) | **Work day: Coefficient −0.64 (−0.76 to −0.53) (P < 0.01)** | ・"We conclude that during a worktime reduction, the total workload of both paid and non-paid work is reduced. The extra free time during a workweek is used for domestic tasks, but also for recovery activities."<br><br>・Subgroup analysis: "The time-use patterns in relation to the intervention **were similar for men and women** as well as for those having children living at home and those who did not."<br>・"Our findings can only be generalized to situations where worktime is reduced and where salary is retained." |
| | | | | | Days off: Coefficient 0.00 (−0.05 to 0.04) | |
| | | | | Work from home (18 months) | Work day: Coefficient −0.03 (−0.07 to 0.01) | |
| | | | | | Days off: Coefficient −0.09 (−0.17 to −0.02) | |
| | | | | Domestic work (18 months) | **Work day: Coefficient 0.20 (0.11 to 0.27) (P < 0.01)** | |
| | | | | | Days off: Coefficient −0.04 (−0.15 to 0.23) | |
| | | | | Child care (18 months) | Work day: Coefficient 0.05 (−0.01 to 0.11) | |
| | | | | | Days off: Coefficient −0.04 (−0.18 to 0.10) | |
| | | | | Care for others (18 months) | Work day: Coefficient 0.01 (−0.01 to 0.03) | |
| | | | | | Days off: Coefficient −0.08 (−0.16 to −0.01) | |
| | | | | Personal care (18 months) | Work day: Coefficient 0.01 (−0.03 to 0.05) | |
| | | | | | Days off: Coefficient −0.01 (−0.08 to 0.05) | |
| | | | | Meals (18 months) | Work day: Coefficient −0.04 (−0.10 to 0.03) | |
| | | | | | Days off: Coefficient 0.00 (−0.06 to 0.07) | |
| | | | | Free-time (18 months) | **Work day: Coefficient 0.10 (0.02 to 0.19)** | |
| | | | | | **Days off: Coefficient 0.28 (0.10 to 0.47) (P < 0.01)** | |

| Study ID | Intervention (Number of participants) | Comparator | Analyses | Outcome (assessment period) | Results (95% Confidence interval) | Comments |
|---|---|---|---|---|---|---|
| | | | | Own-time (18 months) | **Work day: Coefficient 0.20 (0.08 to 0.32) (P < 0.01)** | |
| | | | | | Days off: Coefficient −0.01 (−0.25 to 0.22) | |
| | | | | Socializing (18 months) | Work day: Coefficient 0.06 (−0.02 to 0.13) | |
| | | | | | Days off: Coefficient 0.03 (−0.13 to 0.20) | |
| | | | | Rest (18 months) | Work day: Coefficient −0.03 (−0.11 to 0.05) | |
| | | | | | Days off: Coefficient −0.03 (−0.17 to 0.10) | |
| | | | | Daytime sleep (18 months) | Work day: Coefficient −0.03 (−0.08 to 0.01) | |
| | | | | | Days off: Coefficient −0.05 (−0.12 to 0.02) | |
| | Subsample; social workers (n = 81) (N = 3) | Subsample; social workers (n = 44) (N = 3, one organization was allocated to both conditions due to its size) | (Barck-Holst 2015)<br>・Multiple regression (independent variables: baseline value of measure, group, gender, and age, dependent variable: value of the last measurement point)<br>*B: unstandardized coefficient, *B*: Standardized coefficient | Job demand (18 months) | **B -0.202 (SE 0.089), *B* = -0.183 (P = 0.25)** | "Reduced working hours had a positive effect on restorative sleep, stress, memory difficulties, negative emotion, sleepiness, fatigue and exhaustion both on workdays and weekends; on sleep quality on weekends; and on demands, instrumental manager support and work intrusion on private life." |
| | | | | Job control (18 months) | B 0.060 (SE 0.055), *B* = 0.71 (P = 0.278) | |
| | | | | Social support (18 months) | B -0.016 (SE 0.085), *B* = -0.016 (P = 0.854) | |
| | | | | Instrumental manager support (18 months) | **B 0.425 (SE 0.179), *B* = 0.213 (P = 0.019)** | |
| | | | | Instrumental coworker support (18 months) | B 0.088 (SE 0.143), *B* = 0.045 (P = 0.539) | |
| | | | | Work intrusion on private life (18 months) | **B -0.703 (SE 0.160), *B* = -0.318 (P = 0.000)** | |
| | | | | Private life intrusion on work (18 months) | B -0.021 (SE 0.135), *B* = -0.013 (P = 0.874) | |
| Self-rostering (Flexibility) | | | | | | |
| Albertsen, et al. (2014) [37], Garde, et al. (2012) [38] | Total intervention group (A, B and C): introduction of an IT-planning tool for self-rostering (n = 493 at baseline) (N = 14)<br>Men: about 10% (adjusted for gender)<br>Fathers: n=32 (4%) in both groups | Reference group: no intervention (n = 347 at baseline) (N = 14) | (Albertsen 2014)<br>・Generalized mixed models, Multinomial logistic regression with generalized estimating equations (GEE) (account for within-person associations)<br>・Analyzed the change from baseline to follow-up.<br>・Models included time (baseline and follow-up), intervention (A, B, and C and reference), and the interaction (term time × intervention)<br>・Adjusted for age and gender.<br>*Est.:β-coefficient Estimating | Employee (male and female) | | ・"The effect evaluation of this intervention study showed an overall positive effect of self-rostering on measures of balance between work and private life."<br>・"Positive consequences of self-rostering for recovery and health were observed, particularly in intervention B where worktime control increased but less extensively than intervention"<br>・"The benefits of the intervention were not related to changes in working hours and did not differ by gender, age, family type, degree of employment, or working hour arrangements." |
| | | | | Work-family facilitation (12 months) | OR 1.15 (0.95 to 1.39) (P = 0.153)<br>***Interaction effect between time and intervention status was significant. (P = 0.039)** | |
| | | | | Work-family conflict (12 months) | **Est. -0.085 (-0.16 to -0.01) (P = 0.031)<br>*Interaction effect between time and intervention status was significant. (P = 0.022)** | |
| | | | | Time with children (12 months) | Est. -0.049 (-0.13 to 0.04) (P = 0.256) | |
| | | | | Marital conflicts (12 months) | Est. -0.083 (-0.18 to 0.01) (P = 0.094) | |
| | Intervention A: included a time bank | | (Albertsen 2014) | Work-family facilitation (12 months) | OR 1.35 (0.94 to 1.94) (P = 0.109) | |
| | | | | Work-family conflict (12 months) | Est. -0.075 (-0.22 to 0.07) (P = 0.305) | |

| Study ID | Intervention (Number of participants) | Comparator | Analyses | Outcome (assessment period) | Results (95% Confidence interval) | Comments |
|---|---|---|---|---|---|---|
| | and a puzzle phase (*aim to increase workers satisfaction and well-being) (n = 135 at baseline) (N = 6) | Reference group: no intervention | | Time with children (12 months) | Est. 0.041 (-0.13 to 0.21) (P = 0.630) | |
| | | | | Marital conflicts (12 months) | Est. -0.088 (-0.27 to 0.10) (P = 0.352) | |
| | | | (Garde 2012)<br>*Main effect: odds ratios (OR) or β-coefficient estimating the time × intervention effect. | Influence on working hours | **OR 3.86 (2.34 to 6.37) (P < 0.001)** | |
| | | | | Possibility to request length of duty | **OR 7.07 (4.13 to 12.1) (P < 0.001)** | |
| | | | | Possibility to plan what time of day | **OR 6.43 (3.69 to 11.2) (P < 0.001)** | |
| | | | | Possibility to plan what day to work | **OR 6.96 (3.76 to 12.9) (P < 0.001)** | |
| | | | | Satisfaction with working hours | **OR 2.50 (1.43 to 4.39) (P = 0.001)** | |
| | | | | Consider changing job | OR 0.82 (0.47 to 1.43) (P = 0.477) | |
| | | | | Need for recovery (12 months) | **Est. -0.165 (-0.29 to -0.04) (P = 0.010)** | |
| | | | | Disturbed sleep index (12 months) | Est. 0.081 (-0.076 to 0.23) (P = 0.309) | |
| | | | | Awakening index | Est. 0.072 (-0.083 to 0.23) (P = 0.363) | |
| | | | | Somatic symptoms (12 months) | Est. 0.020 (-0.078 to 0.12) (P = 0.686) | |
| | | | | Mental distress (12 months) | Est. 0.037 (-0.081 to 0.16) (P = 0.542) | |
| | Intervention B: employees could choose what days they wanted to work and not to work and could choose between limited numbers of predefined types of duties (*aim to increase workers satisfaction and well-being) (n = 259 at baseline) (N = 7) | Reference group: no intervention | (Albertsen 2014) | Work-family facilitation (12 months) | **OR 1.36 (1.03 to 1.79) (P = 0.028)** | |
| | | | | Work-family conflict (12 months) | **Est. -0.199 (-0.31 to -0.09) (P < 0.001)** | |
| | | | | Time with children (12 months) | Est. -0.014 (-0.13 to 0.10) (P = 0.813) | |
| | | | | Marital conflicts (12 months) | Est. -0.155 (-0.29 to -0.02) (P = 0.027)<br>*slightly insignificant (P = 0.059) when controlled for work schedule and part time versus full time | |
| | | | (Garde 2012) | Influence on working hours | **OR 2.43 (1.64 to 3.61) (P < 0.001)** | |
| | | | | Possibility to request length of duty | OR 1.27 (0.85 to 1.89) (P = 0.248) | |
| | | | | Possibility to plan what time of day | OR 1.27 (0.83 to 1.93) (P = 0.268) | |
| | | | | Possibility to plan what day to work | **OR 2.48 (1.63 to 3.78) (P < 0.001)** | |
| | | | | Satisfaction with working hours | OR 1.38 (0.90 to 2.12) (P = 0.142) | |
| | | | | Consider changing job | OR 0.68 (0.43 to 1.07) (P = 0.098) | |
| | | | | Need for recovery (12 months) | **Est. -0.170 (-0.27 to -0.065) (P = 0.002)** | |
| | | | | Disturbed sleep index (12 months) | **Est. 0.169 (0.037 to 0.30) (P = 0.012)** | |
| | | | | Awakening index | Est. 0.095 (-0.035 to 0.23) (P = 0.151) | |
| | | | | Somatic symptoms (12 months) | **Est. -0.104 (-0.19 to -0.022) (P = 0.013)** | |
| | | | | Mental distress (12 months) | **Est. -0.131 (-0.23 to -0.032) (P = 0.010)** | |
| | Intervention C: including a time bank | | (Albertsen 2014) | Work-family facilitation (12 months) | **OR 0.65 (0.44 to 0.95) (P = 0.027)** | "Results from the process evaluation suggested that the organizational aim |
| | | | | Work-family conflict (12 months) | **Est. 0.172 (0.01 to 0.34) (P = 0.041)** | |

| Study ID | Intervention<br>(Number of participants) | Comparator | Analyses | Outcome (assessment period) | Results<br>(95% Confidence interval) | Comments |
|---|---|---|---|---|---|---|
| | (employees only choose between a few specific shifts) and a puzzle phase (*aim to optimize the personnel resources)<br>(n = 99 at baseline)<br>(N = 1) | Reference group: no intervention | | Time with children (12 months) | **Est. -0.248 (-0.43 to -0.07) (P = 0.007)** | with the intervention was crucial for the effect." ("whether the primary aim of the implementation is increased flexibility for employees or increased flexibility for employer to optimize staff resources") |
| | | | | Marital conflicts (12 months) | Est. 0.098 (-0.11 to 0.30) (P = 0.344) | |
| | | | (Garde 2012) | Influence on working hours | OR 0.75 (0.46 to 1.23) (P = 0.259) | |
| | | | | Possibility to request length of duty | **OR 2.17 (1.28 to 3.67) (P = 0.004)** | |
| | | | | Possibility to plan what time of day | OR 1.65 (0.93 to 3.92) (P = 0.085) | |
| | | | | Possibility to plan what day to work | **OR 1.89 (1.07 to 3.33) (P = 0.028)** | |
| | | | | Satisfaction with working hours | **OR 0.36 (0.21 to 0.63) (P < 0.001)** | |
| | | | | Consider changing job | **OR 2.93 (1.42 to 6.03) (P = 0.004)** | |
| | | | | Need for recovery (12 months) | Est. 0.037 (-0.101 to 0.18) (P = 0.599) | |
| | | | | Disturbed sleep index (12 months) | Est. 0.141 (-0.032 to 0.31) (P = 0.110) | |
| | | | | Awakening index | Est. 0.042 (-0.128 to 0.21) (P = 0.627) | |
| | | | | Somatic symptoms (12 months) | Est. -0.073 (-0.18 to 0.034) (P = 0.180) | |
| | | | | Mental distress (12 months) | Est. -0.061 (-0.19 to 0.063) (P = 0.356) | |
| Supervisory/ employee training (managing work-family interface) | | | | | | |
| Almeida, et al. (2018) [23], McHale, et al. (2016) [31], Lawson, et al. (2016) [28], McHale, et al. (2015) [32], Davis, et al. (2015) [25], Kelly, et al. (2014) [27], Lee, et al. (2016) [29], | STAR: intervention designed to reduce work-family conflict (N = 27)<br>Men: about 60% (subgroup analysis/ adjusted for gender)<br>Fathers: N/A | Usual practice (UP) (N = 29) | (Olson 2015)<br>・General linear mixed modeling<br>・Analyzed the change from baseline to follow-up.<br>・Key model parameter: interaction between assessment Wave (baseline/ follow-up) and Intervention condition (STAR/ UP).<br>*γ: unstandardized regression coefficient | Employee (male and female) | | ・"The workplace intervention did not overtly address sleep, yet intervention employees slept 8 min/d more and reported greater sleep sufficiency."<br>・"Path models indicated that increased control over work hours and subsequent reductions in work-family conflict mediated the improvement in sleep sufficiency." |
| | | | | Sleep (minimum of 3 valid days of actigraphy) (*main effect: Wave × Intervention interaction)<br>(n = 234 in STAR, n = 240 in UP; participants with ≥3 valid days of actigraphy data) (12 months) | **Total sleep time (per day, min): $\gamma = 8.2$ (0.3 to 16.0)** | |
| | | | | | Wake after sleep onset: $\gamma = 1.3$ (−0.9 to 3.5) | |
| | | | | | **Sleep insufficiency (rating 1-5): $\gamma = -0.2$ (−0.4 to −0.1)** | |
| | | | | | Insomnia symptoms (rating 1-4): $\gamma = -0.0$ (−0.1 to 0.1) | |
| | | | | | Path model analysis: significant direct effects of the intervention on actigraphy-based total sleep time (9min/d), and a significant indirect effect on changes in self-reported sleep insufficiency through increases in control over work hours and subsequent reductions in work-family conflict. | |
| | | | (Lee 2016)<br>・Multilevel models<br>・Analyzed the change from baseline to follow-up.<br>・Adjusted for age, sex, marital/partner status, living with children younger than 18 years in household, providing care to adult relatives, race, and range of current annual household income. | Nighttime/ daytime sleep<br>(*main effect: Wave × Intervention interaction)<br>(n = 195 in STAR, n = 201 in UP; employees who provided valid actigraphy data for 5 to 10 days at the 2 time points) (12 months) | **Daily nighttime sleep duration (min): $B = 8.98$ (SE 3.98) ($p < 0.05$)** | ・"Intervention employees increased nighttime sleep duration at 12 months, by 9 minutes per day, relative to control employees" ("the intervention was particularly effective for older employees (56–70 years) in decreasing their daytime nap duration and day-to-day variability in WASO") |
| | | | | | Daily wake after night sleep onset (WASO) (min): $B = 1.03$ (SE 0.93) ($p > 0.05$) | |
| | | | | | Daily nap duration (min): $B = -4.08$ (SE 4.57) ($p > 0.05$)<br>*There were interaction effects between the intervention and age on daytime nap duration and day-to-day variability in WASO. | |
| | | | (Kelly 2014) | Employee/parents (male and female) | | |

| Study ID | Intervention (Number of participants) | Comparator | Analyses | Outcome (assessment period) | Results (95% Confidence interval) | Comments |
|---|---|---|---|---|---|---|
| Olson, et al. (2015) [33] | | | ・Generalized linear mixed models<br>・Analyzed the change from baseline to follow-up.<br>・Intent-to-treat analysis (regardless how much to participate in the training) | Work-Family Conflict (*main effect: Wave × Intervention interaction) (n = 348 in STAR, n = 346 in UP; employees who completed the six-month follow-up) (*intervention effect for subgroup; 205 men with child at home who completed the six-month follow-up) (6 months) | **Supervisor Support for Family/Personal Life: Est. 0.131 (SE 0.052) (p = 0.015)**<br>***Subgroup: Men with child (subgroup): Supervisor Support for Family/Personal Life: Est. 0.261 (DF 52) (p = 0.009)** | ・"We find statistically significant, though modest, improvements in employees' work-family conflict and family time adequacy and larger changes in schedule control and supervisor support for family and personal life."<br>・"Subgroup analyses suggest the intervention brings greater benefits to employees more vulnerable to work-family conflict." |
| | | | | | **Schedule Control: Est. 0.231 (SE 0.041) (p < 0.001)**<br>***Subgroup: Men with child (subgroup): Work-to-Family Conflict: Est. 0.181 (DF 53) (p = 0.012)** | |
| | | | | | Work-to-Family Conflict: Est. −0.116 (SE 0.060) (p = 0.059)<br>*Subgroup: Men with child (subgroup): Work-to-Family Conflict: Est. −0.189 (DF 53) (p = 0.055) | |
| | | | | | **Family-to-Work Conflict: Est. −0.088 (SE 0.043) (p = 0.045)**<br>*Subgroup: Men with child (subgroup): Family-to-Work Conflict: Est. −0.071 (DF 53) (p = 0.401) | |
| | | | | | **Enough Time for Family: Est. 0.137 (SE 0.059) (p = 0.024)**<br>*Subgroup: Men with child (subgroup): Enough Time for Family: Est. −0.002 (DF 51) (p = 0.986) | |
| | | | | | Psychological Job Demands: Est. −0.075 (p = 0.106)<br>*Subgroup: Men with child (subgroup): Psychological Job Demands: Est. −0.032 (DF 53) (p = 0.710) | |
| | | | (Almeida 2018)<br>・Multilevel models<br>・WP STAR: Within-person level (WP) workplace intervention effects (indicating whether individuals in the STAR condition change outcome at 12 months compared to their baseline assessments)<br>・Analyzed the change from baseline to follow-up.<br>・Adjusted for employees' gender, age, educational level, and race. | Cortisol awakening response (4 consecutive days) (*main effect: WP STAR × type of the day (workdays vs. non-workdays) interaction) (n = 58 in STAR, n = 36 in UP; parents with a child 9–17 years of age) (12 months) | **Est. −2.60 (SE 1.19) (p < 0.05)**<br>(*WP STAR alone was not a significant predictor of CAR: Est. 1.11 (SE 1.23)) | "Although the effect of the intervention was not apparent on average CAR across days, it had an effect on increased CAR on non-workdays." |
| | | | (Davis 2015)<br>・Multilevel models<br>・Analyzed the change from baseline to follow-up.<br>・Intent-to-treat analysis (regardless how much to participate in the training)<br>・Moderated by parent gender, child gender, or child age. | Daily parent-child time (8 consecutive days) (*main effect: Wave ×Intervention interaction) (n = 57 in STAR, n = 36 in UP; parents with a child 9–17 years of age) (12 months) | **Main Effects: *B* = 62.44 (30.76 to 64.13)** | ・"Parents in the STAR intervention reported an average of 39 minutes more time per day or 4.5 hours per week with their child at 1 year after intervention."<br>・ "Changes in parent-child time were more apparent for mothers than for fathers."<br>・ "Although the simple slopes test revealed a trend-level increase in parent-child time for fathers in STAR, |
| | | | | | Moderation Analyses:<br>**Parent gender: *B* = –113.03 (–176.68 to 49.38)**, intervention was more effective in increasing mother-child time than father-child time<br>**Youth gender: *B*= –78.49 (–141.67 to 15.31)**, intervention was more effective in increasing parent-daughter relative to parent-son time | |

| Study ID | Intervention (Number of participants) | Comparator | Analyses | Outcome (assessment period) | Results (95% Confidence interval) | Comments |
|---|---|---|---|---|---|---|
| | | | | | | they did not differ significantly from UP fathers” |
| | | | (McHale 2016)<br>・Multi-level linear regressions<br>・Analyzed the change from baseline to follow-up.<br>・Compare the high attending group (STAR participants who had attended 75% or more of the sessions) to the low attending and to the UP groups.<br>・Adjusted for baseline level of the dependent variable, as well as parent education level and gender, and child age and gender. | Parent-child relationships (child-reported)<br>(*β coefficients: Low attendance vs. High attendance)<br>(n = 125 parent-adolescent dyads, completed baseline and 12-month follow-up home interviews) (12 months) | Parental warmth: Est. −.078 (SE 0.16) ($p > 0.05$) | ・“Results revealed no main effects of the intervention, but children of employees who attended 75% or more program sessions reported more time with their parent and more parent education involvement compared to adolescents whose parents attended less than 75% of sessions” (“the results of intent-to-treat analyses failed to support our hypothesis”) |
| | | | | | **Parents’ education involvement: Est. −0.45 (SE 0.18) ($p < 0.01$)** | |
| | | | | | Parents’ solicitation: Est. −0.25 (SE 0.18) ($p > 0.05$) | |
| | | | | | **Time with parents: Est. −0.33 (SE 0.15) ($p < 0.05$)**<br>(*no evidence of moderation effects by parent gender, youth gender or youth age) | |
| | | | (Lawson 2016)<br>・Multilevel models<br>・Analyzed the change from baseline to follow-up.<br>・The three-way interaction between wave, condition, and stressors also analyzed.<br>・Intent-to-treat analysis<br>・Adjusted for day in study, youth/parent gender, Wave mean school days, youth age, and parents’ annual income. | Child | | “The randomized workplace intervention increased youth positive affect and buffered youth from age-related increases in negative affect and affective reactivity to daily stressors.” |
| | | | | Affective well-being in children (8 consecutive days) (*main effect: Wave × Intervention interaction) (n = 62 in STAR, n = 41 in UP, 9–17 years old children) (12 months) | **Positive affect: *B* = 0.43 (0.32 to 0.55) effect size = 0.30** | |
| | | | | | **Intervention buffered negative association between positive affect and stressors: *B* = 0.68 (0.12 to 1.24), effect size = 0.45**<br>(total number of stressors: between person level) (Wave × Stressor × Condition) | |
| | | | | | **Negative affect: *B* = −0.11 (−0.18 to −0.03), effect size = 0.24** | |
| | | | | | **Intervention buffered positive association between negative affect and stressors: *B* = −0.69 (−1.05 to −0.34), effect size = 0.84**<br>(total number of stressors: between person level) (Wave × Stressor × Condition) | |
| | | | (McHale 2015)<br>・Multilevel models<br>・Analyzed the change from baseline to follow-up.<br>・Adjusted for youth age and gender, day in study, and Wave mean school days. | Employee/parents (male and female) and child | | “Our findings showed that a workplace intervention (…) had corollary effects on the sleep of employees’ adolescent-aged offspring.” |
| | | | | Sleep in child (8 consecutive days) (*main effect: Wave ×Intervention interaction)<br>(n = 57 in STAR, n = 36 in UP, 9–17 years old children) (12 months) | Sleep Duration: Est. −0.07 (SE 0.15) ($p > 0.05$) | |
| | | | | | **Sleep Variability: Est. −0.26 (SE 0.13) ($p < 0.05$)** | |
| | | | | | **Sleep Latency: Est. −0.27 (SE 0.06) ($p < 0.001$)** | |
| | | | | | **Sleep Quality: Est. 0.15 (SE 0.05) ($p < 0.01$)** | |
| Workplace parenting intervention (managing work-family interface) | | | | | | |
| Haslam, et al. | Workplace Triple P (a workplace parenting intervention); aimed | Waitlist control (n = 43, analyzed at | ・ANCOVA, MANCOVA (with post-intervention scores as dependent variables and pre- | Employee/parents (male and female) | | ・“All major hypotheses were supported.” (“The only variable that |
| | | | | Work stress (3 weeks) | **Overall work stress: $F(1, 80) = 9.9885$ ($p = 0.002$), Cohen’s d = 0.7 (0.08 to 0.79), MANCOVA: $F(2, 82) = 8.509$ ($p < 0.001$)** | |

| Study ID | Intervention (Number of participants) | Comparator | Analyses | Outcome (assessment period) | Results (95% Confidence interval) | Comments |
|---|---|---|---|---|---|---|
| (2013) [26] | at reducing work–family conflict and improving work and family functioning in teachers. (n = 43, analyzed at post-intervention) Men: about 20-30% Fathers: all male participants had children aged 2-12 years living at home | post-intervention) | intervention scores included as covariates) | | **(related variables: student behavior stress and workload-related stress)** | did not change as a result of the intervention was job satisfaction") ·"Intervention effects were maintained at 4-month follow-up." ·"The results indicate that a parenting intervention can reduce work-family conflict and occupational stress and improve family functioning in teachers balancing work and family." |
| | | | | | **Student-related stress: F (1, 86) = 13.658 (p = 0.000), Cohen's d = 0.60 (0.17 to 1.02)** | |
| | | | | | **Task overload stress factor: F (1, 86) = 13.310 (p = 0.000), Cohen's d = 0.47 (0.05 to 0.89)** | |
| | | | | Depression and anxiety (3 weeks) | **DASS depression: F (1, 84) = 7.405 (p = 0.008), Cohen's d = 0.58 (0.21 to 1.06)** | |
| | | | | | **DASS anxiety: F (1, 84) = 7.099 (p = 0.009), Cohen's d = 0.57 (0.46 to 1.33)** | |
| | | | | | **Parental adjustment: F (2, 84) = 4.588 (p = 0.013) (MANCOVA, related variables: anxiety and depression)** | |
| | | | | Work family conflict (3 weeks) | **Family-to-work conflict: F (1, 86) = 8.345 (p = 0.005), Cohen's d = 0.62 (0.08 to 0.78)** | |
| | | | | | **Work-to-family conflict: F (1, 86) = 7.744 (p = 0.007), Cohen's d = 0.60 (0.04 to 0.89)** | |
| | | | | Job satisfaction (3 weeks) | F (1, 85) = 1.773 (p = 0.186), Cohen's d = 0.19 (0.025 to 0.58) | |
| | | | | Parental satisfaction (3 weeks) | **F (1, 80) = 7.119 (p = 0.009), Cohen's d = 0.59 (0.12 to 1.0)** | |
| | | | | Dysfunctional parenting (3 weeks) | **Laxness: F (1, 74) = 12.590 (p = 0.001), Cohen's d = 0.76 (0.08 to 0.93)** | |
| | | | | | **Over reactivity: F (1, 74) = 20.494 (p = 0.001), Cohen's d = 0.97 (0.40 to 1.27)** | |
| | | | | | **Verbosity: F (1, 74) = 12.665 (p = 0.001), Cohen's d = 0.76 (0.27 to 1.12)** | |
| | | | | | **Dysfunctional parenting style: F (3, 82) = 7.555 (p = 0.001) (MANCOVA, related variables: ECBC intensity and ECBC problems)** | |
| | | | | Parenting efficacy (3 weeks) | **Behavior (dealing with different types of behavior problems): F (1, 74) = 15.661 (p = 0.000), Cohen's d = 0.91 (0.16 to 1.1)** | |
| | | | | | Setting (dealing with behavior across different home and community settings): F (1, 74) = 5.195 (p = 0.026), Cohen's d = 0.52 (0.11 to 0.78) (*Bonferroni adjustment =.025) | |
| | | | | | **Parenting efficacy: F (2, 73) = 7.729 (p = 0.001) (MANCOVA, related variables: behavior and setting management efficacy)** | |
| | | | | Teaching-related self-efficacy (3 weeks) | **F (1, 85) = 10.215 (p = 0.002), Cohen's d = 0.69 (0.13 to 0.98)** | |
| | | | | Child | | |

| Study ID | Intervention (Number of participants) | Comparator | Analyses | Outcome (assessment period) | Results (95% Confidence interval) | Comments |
|---|---|---|---|---|---|---|
| | | | | Problem behavior (child) (3 weeks) | **ECBI Intensity (frequency of disruptive behaviors): F (1, 74) = 13.22 (p = 0.001), Cohen's d = 0.83 (0.04 to 0.86)** | |
| | | | | | **ECBI Problem (number of disruptive behaviors): F (1, 74) = 11.6 (p = 0.001), Cohen's d = 0.78 (0.05 to 0.95)** | |
| | | | | | **Disruptive behavior: F (2, 73) = 7.913 (p = 0.001) (MANCOVA, related variables: ECBC intensity and ECBC problems)** | |
| Martin and Sanders (2003) [30 | Workplace Triple P (a group version of the Triple-P Positive Parenting Program (WPTP) designed specifically for delivery in the workplace) (n = 16, analyzed at post-intervention) Fathers: all male participants had children aged 2-9 years living at home | Waitlist control (n = 11, analyzed at post-intervention) | ・ANCOVA (with post-intervention scores as dependent variables and pre- intervention scores included as covariates) | Employee/parents (male and female) | | ・"Following intervention, parents in WPTP reported significantly lower levels of disruptive child behavior, dysfunctional parenting practices, and higher levels of parental self-efficacy in managing both home and work responsibilities, than parents in the WL condition." ・"These short-term improvements were maintained at 4-months follow-up." |
| | | | | Work stress (8 weeks) | F (1, 27) = 0.67 (p = 0.42) | |
| | | | | Parental adjustment (DASS) (8 weeks) | F (1, 27) = 0.58 (p = 0.45) | |
| | | | | Dysfunctional parenting (8 weeks) | **F (1, 27) = 7.40 (p < 0.01)** | |
| | | | | Home efficacy (PSBC) (8 weeks) | **F (1, 27) = 11.62 (p < 0.01)** | |
| | | | | Job satisfaction (8 weeks) | F (1, 27) = 0.09 (p = 0.77) | |
| | | | | Social support (8 weeks) | F (1, 27) = 0.37 (p = 0.55) | |
| | | | | Work efficacy (PSBC) (8 weeks) | **F (1, 27) = 11.30 (p =<0.01)** | |
| | | | | Work commitment (8 weeks) | F (1, 27) = 0.28 (p = 0.60) | |
| | | | | Child | | |
| | | | | Problem behavior (child) (8 weeks) | **ECBI intensity: F (1, 27) = 5.76 (p = 0.02)** | |
| | | | | | **ECBI problem: F (1, 27) = 4.25 (p = 0.05)** | |
| Sanders, et al. (2011) [34] | Workplace Triple P (consisted of two components: work-family balance coping skills and positive parenting skills) (n = 48, analyzed at post-intervention) Fathers: all male participants had children aged 1-16 years living at home | Waitlist control (n = 52, analyzed at post-intervention) | ・ANCOVA (with pre-intervention scores used as covariates) ・Missing data: The last observation carried forward (LOCF) procedure was utilized in measures of the DASS, PS, and SDQ scales (at post-intervention assessment). | Employee/parents (male and female) | | "Results showed that parents who had received the intervention reported significantly lower levels on measures of personal distress and dysfunctional parenting; and higher levels of work commitment, work satisfaction, and self-efficacy." |
| | | | | Parental distress (DASS) (8 weeks) | **Anxiety: F (1, 95) = 5.23 (p = 0.024), Cohen's d = 0.48** | |
| | | | | | Depression: F (1, 95) = 2.20 (p = 0.14), Cohen's d = 0.33 | |
| | | | | | **Stress: F (1, 95) = 16.37 (p < 0.01), Cohen's d = 0.83** | |
| | | | | | **Total: F (1, 95) = 10.40 (p = 0.002), Cohen's d = 0.64** | |
| | | | | Dysfunctional parenting (Parenting scale) (8 weeks) | Laxness: F (1, 95) = 3.69 (p = 0.06), Cohen's d = 0.41 | |
| | | | | | **Over-reactivity: F (1, 95) = 7.54 (p = 0.008), Cohen's d = 0.12** | |
| | | | | | **Verbosity: F (1, 95) = 6.64 (p = 0.012), Cohen's d = 0.21** | |
| | | | | | **Total: F (1, 95) = 12.39 (p = 0.001), Cohen's d = 0.40** | |
| | | | | Work satisfaction (8 weeks) | **F (1, 93) = 4.01 (p = 0.048), Cohen's d = 0.57** | |
| | | | | Work stress (8 weeks) | F (1, 93) = 2.80 (p = 0.10), Cohen's d = 0.36 | |
| | | | | Work commitment (8 weeks) | **Union: F (1, 93) = 6.44 (p = 0.01), Cohen's d = 0.36** | |
| | | | | | Organization: F (1, 93) = 0.266 (p = 0.61), Cohen's d = 0.10 | |

| Study ID | Intervention (Number of participants) | Comparator | Analyses | Outcome (assessment period) | Results (95% Confidence interval) | Comments |
|---|---|---|---|---|---|---|
| | | | | | Occupation: F (1, 93) = 2.81 (p = 0.10), Cohen's d = 0.05 | |
| | | | | | Job: F (1, 93) = 0.36 (p = 0.55), Cohen's d = 0.09 | |
| | | | | Parenting efficacy (Parenting Task Checklist) (8 weeks) | **F (1, 93) = 6.93 (p = 0.01), Cohen's d = 0.37** | |
| | | | | Child | | |
| | | | | Child behavior problem (8 weeks) | ECBC Intensity: F (1, 60) = 2.23 (p = 0.14), Cohen's d = 0.27 | |
| | | | | | ECBC Problem: F (1, 60) = 3.37 (p = 0.06), Cohen's d = 0.16 | |
| | | | | | SDQ (Total): F (1, 60) = 0.52 (p = 0.47), Cohen's d = 0.11 | |
| Individualized counseling to employees (experiencing personal and work-related difficulties) | | | | | | |
| Nunes, et al. (2018) [39], Richmond, et al. (2016) [40], Richmond, et al. (2017) [41] | Employee Assistance Programs (EAPs): offer individualized counseling to employees that support employees to identify effective coping strategies for personal and professional stressors. Men: about 30% | Non-EAP users | (Nunes 2017)<br>・Repeated-measures mixed models<br>・Product term: time × condition<br>・Account of the skewed distribution of work time lost: log of work hours lost, count of work hours lost, divide work hours lost into three categories (low, moderate, high).<br>・Analyzed the change from baseline to follow-up.<br>・Control for the outcome measure before baseline and the baseline survey date.<br>・Intent-to-treat design (regardless of whether or not they eventually received services) | Employee (male and female) | | ・"EAP users on average experienced a significantly greater reduction of sick leave hours than those who did not seek EAP services."<br>・"this study provides empirical evidence that EAP services are ef-fective at reducing absenteeism among the population they seek to serve: those suffering from mild-to-moderate prob-lems."<br>・"Work-based, one-on-one counseling services through EAPs offer employees confidential, easy-to-access services that benefit employees and employers alike." |
| | | | | Sick leave usage (n = 145 in EAP, n = 145 in control) (12 months) | **Logged sick hours (Month × EAP): Est. -0.049, t value -2.36 (p < 0.05)**, (EAP): Est. -0.311, t value -1.68 (p > 0.05) (4.8 % fewer hours lost per month than the control group)<br>***The linear decline of sick hours with time is steeper for the EAP participants than the control participants.** | |
| | | | | | **Sick hours count (Month × EAP): Est. -0.067, t value -2.35 (p < 0.05)**, (EAP): Est. -0.368, t value -1.25 (p > 0.05) (6.5 % fewer hours lost per month than the control group) | |
| | | | | | **0-3 work hours lost (no more than 1 week per year) versus 4+ work hours lost (Month × EAP): Est. -0.104, t value -2.91 (p < 0.01), (EAP): Est. -0.652, t value -2.03 (p < 0.05)** | |
| | | | | | 0-7 work hours lost (no more than 2 weeks per year) versus 8+ hours lost: Est. -0.066, t value -1.81 (p > 0.05), (EAP): Est. -0.321, t value -0.99 (p > 0.05)<br>("The program appears to help those with problems producing moderate use of sick leave more so than those with problems leading to chronically high sick leave.") | |
| | | | (Richmond 2017)<br>・Linear regression models (for scale outcome of presenteeism and workplace distress) and negative binomial regression (for count outcome of absenteeism)<br>・Analyzed the change from baseline to follow-up.<br>・Four moderators: the pretest outcome, hazardous alcohol use, depression, and anxiety.<br>*Est.:β-coefficient Estimating | Presenteeism (*main effect: EAP, controlling for baseline outcomes) (n = 156 in EAP, n = 188 in control) (2 to 12 months, average: 5.98 months) | **Est. -0.10, t value -2.08 (p < 0.05)** | ・"Positive associations were detected for both reductions in absences from the worksite and improvements in productivity when at work."<br>・"EAP did more to reduce absenteeism for those who began with lower severity of depression and anxiety at baseline. |
| | | | | Absenteeism (*main effect: EAP, controlling for baseline outcomes) (n = 156 in EAP, n = 188 in control) (2 to 12 months, average: 5.98 months) | **Est. 0.55, t value 11.3 (p < 0.01) *** negative binomial regression<br>***Tests for moderation (there are two statistically significant interactions for absenteeism; pretest depression and anxiety) indicates that the treatment does more to reduce absenteeism for those who begin with lower severity at pretest.** | |
| | | | | Workplace distress (*main effect: EAP, controlling for baseline outcomes) (n = 156 in EAP, n = 188 in control) (2 to 12 months, average: 5.98 months) | Est. -0.03, t value -0.76 (p > 0.05) | |

| Study ID | Intervention<br>(Number of participants) | Comparator | Analyses | Outcome (assessment period) | Results<br>(95% Confidence interval) | Comments |
|---|---|---|---|---|---|---|
| | | | (Richmond 2016)<br>・Linear regression models (with the treatment and pretest outcomes as predictors.) (negative binomial regression for absenteeism)<br>・Sobel test to evaluate the significance of the indirect treatment effects via the mediators; demonstrated the ability of an EAP to improve work performance via improvements in depression and anxiety.<br>*b: unstandardized coefficient, *b*: Standardized coefficient | PHQ-8: depression (n = 338) (2 to 12 months, average: 5.98 months) | **b -1.098 (SE 0.463), *b* = -0.100; *t* = -2.37 (-2.009 to -0.187)** | ・"EAP significantly reduced symptoms of depression and anxiety, but not at risk alcohol use."<br>・"EAP reductions in depression and anxiety mediated EAP-based reductions in absenteeism and presenteeism." (Sobel test) |
| | | | | GAD-2: anxiety (n = 339) (2 to 12 months, average: 5.98 months) | **b -0.327 (SE 0.158), *b* = -0.092; *t* = -2.06 (-0.638 to -0.016)** | |
| | | | | AUDIT: alcohol (n = 340) (2 to 12 months, average: 5.98 months) | b 0.069 (SE 0.238), *b* = -0.009; *t* = 0.29 (-0.399 to 0.537) | |
| | | | | Workplace distress | Mean in EAP 2.69 (SD 1.1), mean in comparison 2.66 (SD 1.2) | |
| | | | | EAP Effect on Presenteeism Without and with Clinical Mediating Variables (n=338) | Depression<br>**Without: b -0.213 (SE 0.105), *b* = -0.095; *t* = -2.03 (P < 0.05)**<br>With: b -0.158 (SE 0.099), *b* = -0.071; *t* = -1.60 (P > 0.05) | |
| | | | | | Anxiety<br>**Without: b -0.213 (SE 0.105), *b* = -0.095; *t* = -2.03 (P < 0.05)**<br>With: b -0.162 (SE 0.100), *b* = -0.072; *t* = -1.62 (P > 0.05) | |
| | | | | | Alcohol<br>**Without: b -0.213 (SE 0.105), *b* = -0.095; *t* = -2.03 (P < 0.05)**<br>**With: b -0.213 (SE 0.105), *b* = -0.095; *t* = -2.04 (P < 0.05)** | |
| | | | | EAP Effect on (Log of) Absenteeism Without and with Clinical Mediating Variables (n=338) | Depression<br>**Without: b -0.448 (SE 0.138), *b* = -0.16; *t* = -3.24 (P < 0.01)**<br>**With: b -0.420 (SE 0.135), *b* = -0.151; *t* = -3.11 (P < 0.01)** | |
| | | | | | Anxiety<br>**Without: b -0.448 (SE 0.138), *b* = -0.16; *t* = -3.24 (P < 0.01)**<br>**With: b -0.420 (SE 0.135), *b* = -0.151; *t* = -3.11 (P < 0.01)** | |
| | | | | | Alcohol<br>**Without: b -0.448 (SE 0.138), *b* = -0.16; *t* = -3.24 (P < 0.01)**<br>**With: b -0.448 (SE 0.138), *b* = -0.161; *t* = -3.25 (P < 0.01)** | |

Bold indicates significance at P < 0.05 level; N/A means information not available in included study report or from study authors at the time of submitting this review
